# Interaction Between the Complement System and Infectious Agents – A Potential Mechanistic Link to Neurodegeneration and Dementia

**DOI:** 10.3389/fncel.2021.710390

**Published:** 2021-08-02

**Authors:** Noriko Shinjyo, Wataru Kagaya, Marcela Pekna

**Affiliations:** ^1^Laboratory of Immune Homeostasis, WPI Immunology Frontier Research Center, Osaka University, Osaka, Japan; ^2^School of Tropical Medicine and Global Health, Nagasaki University, Nagasaki, Japan; ^3^Department of Parasitology and Research Center for Infectious Disease Sciences, Graduate School of Medicine, Osaka City University, Osaka, Japan; ^4^Laboratory of Regenerative Neuroimmunology, Center for Brain Repair, Department of Clinical Neuroscience, Institute of Neuroscience and Physiology, Sahlgrenska Academy at the University of Gothenburg, Gothenburg, Sweden; ^5^Florey Institute of Neuroscience and Mental Health, Parkville, VIC, Australia

**Keywords:** complement, infection, dementia, Alzheimer’s disease, inflammation

## Abstract

As part of the innate immune system, complement plays a critical role in the elimination of pathogens and mobilization of cellular immune responses. In the central nervous system (CNS), many complement proteins are locally produced and regulate nervous system development and physiological processes such as neural plasticity. However, aberrant complement activation has been implicated in neurodegeneration, including Alzheimer’s disease. There is a growing list of pathogens that have been shown to interact with the complement system in the brain but the short- and long-term consequences of infection-induced complement activation for neuronal functioning are largely elusive. Available evidence suggests that the infection-induced complement activation could be protective or harmful, depending on the context. Here we summarize how various infectious agents, including bacteria (e.g., *Streptococcus* spp.), viruses (e.g., HIV and measles virus), fungi (e.g., *Candida* spp.), parasites (e.g., *Toxoplasma gondii* and *Plasmodium* spp.), and prion proteins activate and manipulate the complement system in the CNS. We also discuss the potential mechanisms by which the interaction between the infectious agents and the complement system can play a role in neurodegeneration and dementia.

## Introduction

### The Complement System

**FIGURE 1:**
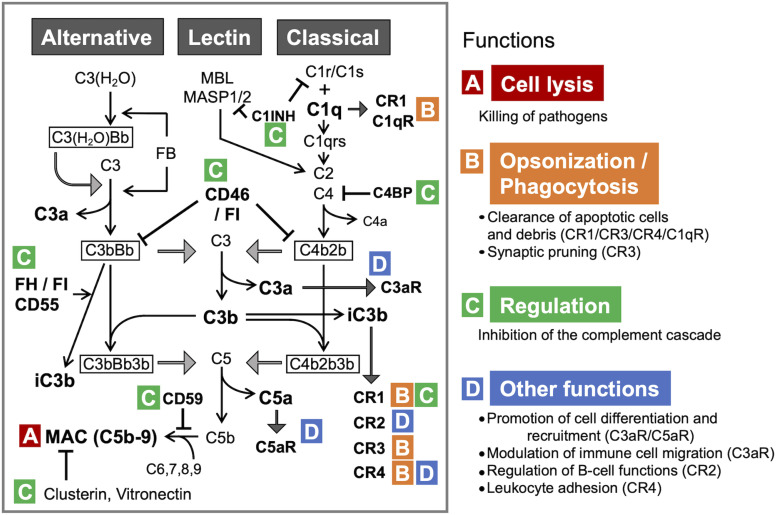
The complement system: activation pathways and functions in the CNS. All three pathways converge at C3. The formation of C3 convertase [C3(H_2_O)Bb and C3bBb in the alternative pathway or C4b2b in the classical and lectin pathways], followed by C3 cleavage, leads to the formation of MAC, the terminal complement complex **(A)**. C1q and iC3b also facilitate immune responses via opsonization and promoting phagocytosis through binding to the receptors (CR1 and C1qR for C1q; CR1, CR2, and CR3 for iC3b) **(B)**. Under physiological conditions, complement activation is tightly controlled by the regulators of complement activation (RCAs) **(C)**. C3a and C5a, the smaller fragments generated through the proteolytic activation of C3 and C5, respectively, regulate differentiation and mobilization of immune and non-immune cells through the cognate receptors C3aR and C5aR, respectively **(D)**. C3 and C5 can be also cleaved by other proteases via the so-called extrinsic pathway. C1qR, C1q receptor; C3bBb3b, C5 convertase formed by binding of additional C3b to C3bBb (C3 convertase); C3(H_2_O), hydrated C3; C3(H_2_O)Bb, the initiating C3 convertase of alternative pathway, consisting of C3(H_2_O) and Bb (Factor B [FB] cleavage product); CR1, complement receptor type 1; CR2, complement receptor type 2; CR3, complement receptor type 3; CR4, complement receptor type 4; FH, complement factor H; FI, complement factor I. C4BP, C4-binding protein; C1INH, C1-inhibitor.

The complement system is an evolutionarily conserved proteolytic cascade, consisting of over 40 components (Schartz and Tenner, 2020). Complement plays a key role as part of the innate immunity, eliminating pathogens and damaged cells, directly killing bacteria by forming the so-called membrane attack complex (MAC), and promoting inflammatory responses via the generation of anaphylatoxins (Yanamadala and Friedlander, 2010). The activation of the complement system can be triggered via three pathways: the classical pathway, the lectin pathway, and the alternative pathway (Veerhuis et al., 2011; Figure 1).

Each of the activation pathways leads to the formation of C3 convertase (C4bC2b or C3bBb) that cleaves the third complement component (C3) into the C3a and C3b fragments, the latter of which functions as an opsonin, forms the C3 convertase of the alternative pathway (C3bBb) and takes part in the next step of the activation cascade, i.e., proteolytic cleavage of C5. C5 activation results in the release of C5a and the formation of MAC, a.k.a. the terminal complement complex, C5b-9. C3a and C5a are peptides with anaphylatoxin properties and function as fluid-phase inflammatory mediators. They exert their biological effects mainly through the cognate membrane-bound receptors C3a receptor (C3aR) and C5a receptor (C5aR1), respectively. C5a can also bind to the second C5a-like receptor (C5L2, C5aR2). C5aR1 can propagate proinflammatory signals to induce cytokine/chemokine release and mediate the chemoattraction and activation of neutrophils and macrophages (Mathern and Heeger, 2015). C3aR signaling is immunomodulatory, exerting pro- or anti-inflammatory effects in a context-dependent manner (Coulthard and Woodruff, 2015). For example, C3aR activation enhances phagocytosis by macrophages (Wu et al., 2019) and suppresses injury-induced mobilization of neutrophils (Wu et al., 2013; Brennan et al., 2019). Besides its C3aR-mediated actions, C3a has a direct anti-microbial activity (Pasupuleti et al., 2007), indicating its multiple modes of action as an anti-infective agent. Even without antibodies, complement can neutralize pathogens (Speth et al., 2002). In addition, there are links between complement and adaptive immunity, such as the robust augmentation of antibody responses mediated by the C3d fragment (proteolytically generated from C3b) and complement receptor 2 (CR2) (Dempsey et al., 1996) and the regulation of T cell activation by C3a/C5a (Lalli et al., 2008). Thus, the complement system has crucial functions in both innate and adaptive immune responses (Mathern and Heeger, 2015). In addition, recent reports show that a number of cell types store intracellular C3 (Elvington et al., 2016), which has been proposed to have an important role in the regulation of cellular homeostasis (Liszewski et al., 2017). For example, cathepsin L-mediated intracellular C3 cleavage and C3a generation are required for homeostatic T cell survival (Liszewski et al., 2013). Airway epithelial cells can *de novo* synthesize C3, and intracellular C3 protects these cells from stress-induced cell death (Kulkarni et al., 2019). Intracellular C3 is also required for autophagic turnover of pancreatic β cells during diabetic stress (King et al., 2019). Notably, C3 is an evolutionarily ancient protein; it is considered to be the first element of the original complement system, whose primary role was to guard intracellular environment and homeostasis from various stimuli (Elvington et al., 2016). While complement is widely acknowledged as part of vertebrate humoral immunity, the significance of intracellular complement is less recognized. The potential role for intracellular C3 in self-defense against infection warrants further investigation.

The classical pathway is initiated by binding of C1 (C1qrs) to activators such as immune complexes, microbes, apoptotic cells, and some specific proteins including fibrillar amyloid β (Aβ) (Veerhuis et al., 2011). The lectin pathway is activated by binding of mannose-binding lectin (MBL) and ficolin to carbohydrates on microorganisms or dying cells (Veerhuis et al., 2011). The classical and lectin pathways converge at the proteolytic cleavage of C4 and C2 and the formation of C4bC2b, the C3 convertase shared by these pathways. The alternative pathway continuously generates low levels of C3-derived activation products and C3 convertase C3bBb, providing a natural immunity to microbes (Farriesa and Atkinson, 1991). The activation of alternative pathway can also occur as a result of direct interaction between C3b and pathogenic molecules, including fibrillar Aβ, further amplifying the downstream of complement cascade (Tenner, 2020). C3 and C5 can be cleaved also by MASP 1 (Matsushita and Fujita, 1995), neutrophil elastase (Yuan et al., 2015), cathepsins (Liszewski et al., 2013; Yuan et al., 2015), granulocyte proteases (Johnson et al., 1976), lysosomal enzymes, kallikrein, coagulation factors XIa, Xa, IXa, thrombin, and plasmin (Markiewski and Lambris, 2007; Amara et al., 2010). This non-convertase mode of activation is also called the extrinsic pathway.

Under physiological conditions, complement activity is tightly regulated by regulators of complement activation (RCA) to safeguard against autologous tissue injury. The RCAs that limit complement activation both in time and space include factor H (FH), factor I, C1-inhibitor, C4-binding protein, clusterin, vitronectin, CD46 (membrane cofactor protein, MCP), CD55 (decay accelerating factor, DAF), and complement receptor 1 (CR1, CD35). Properdin (FP), on the other hand, acts as a positive regulator that stabilizes the alternative pathway C3-convertase (Figure 1).

### The Complement System in the CNS

Although the liver is the major source of circulating soluble complement proteins (Perlmutter and Colten, 1986), it is now established that complement components are locally synthesized throughout the body, including the brain (Woodruff et al., 2010; Tenner et al., 2018). Complement proteins, including the regulatory factors and receptors, are produced by glia and neurons (Gasque et al., 1998; Ischenko et al., 1998; Nataf et al., 1999), and recent studies have revealed a range of non-immune functions of the complement system in the CNS. For example, complement deposition on synapses, followed by CR3-dependent microglial engulfment and elimination of C1q- and C3b/iC3b-tagged synapses, plays a crucial role in synaptic pruning during normal development (Schafer et al., 2012). C3aR and C5aR are expressed by neurons and glia in the brain under normal and pathological conditions (Barnum et al., 2002). C3aR signaling regulates neuronal migration during CNS development (Gorelik et al., 2017), controls neural progenitor cell proliferation (Coulthard et al., 2018), differentiation and migration *in vitro* (Shinjyo et al., 2009), and promotes basal as well as injury-induced neurogenesis in the adult mouse brain (Rahpeymai et al., 2006; Shinjyo et al., 2009; Stokowska and Pekna, 2018). Notably, mice lacking C3 exhibit excessive number of synapses as young adults (Stevens et al., 2007; Perez-Alcazar et al., 2014) and neuronal migration defects characteristic of autism spectrum disorder (Gorelik et al., 2017), and are protected against age-related loss of synapses in the hippocampus (Shi et al., 2015). Higher expression of C4 in the brain has been implicated in excessive loss of synapses and development of schizophrenia (Sekar et al., 2016). C3aR deficiency is associated with altered morphology of the hippocampus and amygdala, mild cognitive impairment and hyperactive behavior in unchallenged mice (Coulthard et al., 2018; Pozo-Rodrigálvarez et al., 2021) as well as reduced neural plasticity responses after ischemic brain injury (Stokowska et al., 2017). Thus, the complement system is required for the normal development and function of the CNS.

Considering the broad range of neurodevelopmental and neuroregulatory functions of complement in the brain, it is not surprising that dysfunction or aberrant activation of the complement system have been implicated in the pathogenesis of a number of brain disorders (Gasque et al., 2000; Morgan, 2015). More specifically, complement activation has been observed in human neurodegenerative diseases, such as Alzheimer’s disease (AD), prion disease, chromosome 13 dementias (Eikelenboom et al., 2002; Rostagno et al., 2002; Morgan, 2018), as well as other neurological disorders associated with the blood-brain barrier (BBB) dysfunctions such as cerebrovascular disease, multiple sclerosis (MS) (Storch et al., 1998), and meningitis (Koelman et al., 2019). In infection, while providing protection against pathogens, excessive or prolonged activation of the complement system can lead to exaggerated inflammation and unfavorable outcomes (Ross and Densen, 1984; Figure 1).

### Complement and Dementia

Dementia is a broad term for conditions with progressive memory impairment, executive and behavioral anomaly, and emotional problems, affecting the ability to perform day-to-day activities. Complement activation is implicated in AD, the most prevalent form of dementia (Rubio-Perez and Morillas-Ruiz, 2012). Together with glial activation and pro-inflammatory cytokine release, complement activation occurs during the early stage of the disease progression (Aiyaz et al., 2012). As described above, amyloid proteins stimulate complement activity (Veerhuis et al., 2011; Tenner, 2020), leading to the activation of the surrounding glial cells and local chronic inflammation (Cadman and Puttfarcken, 1997; Veerhuis et al., 2011). Complement proteins are present in amyloid plaques in the brain (Aiyaz et al., 2012), and co-localize with Aβ in the brain capillaries of cerebral amyloid angiopathy (Matsuo et al., 2017), suggesting that complement activation is a key event associated with amyloid deposition. In addition, complement is likely involved in other forms of dementia, including chromosome 13 dementias (familial British dementia FBD and familial Danish dementia FDD). Similar to AD, chromosome 13 dementias are associated with neurodegeneration and amyloid deposition in the CNS. In spite of the structural differences in the disease-associated amyloid proteins (Aβ in AD, ABri in FBD, ADan in FDD), neurodegeneration and amyloid deposition, associated with glial activation and local inflammation, are the common hallmark in all of these dementias (Saul et al., 2013). Importantly, complement activation products co-localize with amyloid plaques and related deposits in FBD and FDD (Rostagno et al., 2002). ABri and ADan synthetic peptides activate classical and alternative complement pathways, leading to the formation of the terminal complex (C5b-9). These data suggest that chronic complement activation associated with amyloid deposits may be a contributing factor to dementia progression in chromosome 13 dementias as well as in AD. While experimental evidence points to the critical function of complement in Aβ clearance (Wyss-Coray et al., 2002), and C3- and CR3-dependent microglial phagocytosis appears to play a beneficial role in the elimination of foreign pathogens as well as Aβ (Fu et al., 2012), CR3 also limits Aβ clearance from the brain interstitial fluid (Czirr et al., 2017), and is involved in Aβ-induced microglia-mediated loss of synapses in AD (Hong et al., 2016). The complexity of the involvement of complement in AD type of neurodegeneration is further highlighted by findings in the C3 deficient mice carrying the AD-associated mutations in the amyloid polypeptide and presenilin 1 genes. In this model, the C3 deficiency was protective against plaque-related synapse and neuron loss as well as cognitive decline, despite higher plaque burden (Shi et al., 2017). In addition, C1q and CR3 are required for the clearance of neuronal debris by microglia, at least in the context of injury-induced neurodegeneration (Norris et al., 2018). By virtue of its proinflammatory functions, C5a plays a significant role in neurodegeneration, including AD (Landlinger et al., 2015). Jointly, these reports point to the role of the complement system in Aβ and debris clearance as well as synapse elimination, Aβ-induced neurodegeneration and dementia.

### Complement and Brain Infection

In the presence of an intact BBB, circulating immune cells, such as B- and T-lymphocytes, have limited access to the CNS. As a result, the defense of brain tissue against invading pathogens is largely dependent on local innate immunity, in particular the complement system. A number of pathogens escape the peripheral immune responses and enter the CNS. The interaction between pathogens and complement can lead to the clearance of pathogens, however, it may also contribute to tissue damage. Here we summarize the interactions between complement and infectious organisms, including bacteria, viruses, fungi, and parasites (Table 1), and discuss the potential impact of these interactions on CNS function. The interactions between complement and prion proteins are also discussed.

## Interactions Between Infectious Agents and Complement in the CNS

### Bacteria

Genetic defects in the complement system increase the susceptibility to bacterial infection (Brouwer et al., 2009). C2, factor D (FD), and FP deficiencies are associated with predisposition to meningococcal disease (Fijen et al., 1999; Jönsson et al., 2005; Sprong et al., 2006), while single-nucleotide polymorphisms (SNPs) in MBL are associated with pneumococcal disease (Brouwer et al., 2009). FH genotype 496C/C is associated with meningococcal disease (Haralambous et al., 2006) as is a locus in the FH (*CFH*) region (Davila et al., 2010). C3a exhibits anti-microbial activity against Gram-negative and Gram-positive bacteria (Pasupuleti et al., 2007). These data show that complement-dependent immune response is crucial in the protection against bacterial infection.

The evidence for the involvement of the complement system in bacterial meningitis is summarized in Table 1.1. Bacterial meningitis is an acute inflammation caused by infection in the meninges that envelope and protect the brain and spinal cord. Commonly caused by *Streptococcus pneumoniae*, *Neisseria meningitidis*, and *Listeria monocytogenes*, bacterial meningitis is a major health threat, the morbidity and mortality of which are driven by dysregulated host immune reactions (Mook-Kanamori et al., 2011; van de Beek et al., 2016).

**TABLE 1.1:** Interactions between complement and bacteria in the CNS.

	Pathogen	Study design	Outcomes	Conclusion(s)	References
1	*Staphylococcus epidermidis*	(*in vivo*) Neonatal infection and sepsis model in preterm pigs, induced by systemic infection with *S. epidermidis.*	(1) *S. epidermidis* inoculation caused sepsis and blood CSF barrier disruption in preterm pigs. (2) Acute-phase immune response proteins, including complement proteins (**C1r**, **C3**, and **C5**), were up-regulated in CSF at 24 h post-infection.	*S. epidermidis* systemic infection increased the levels of **C1r**, **C3**, and **C5** in the CSF.	Muk et al., 2019
2	*Streptococcus suis*	(1) (*in vitro*) Human BBB model using hCMEC/D3 and *S. suis* (WT, Fhb deficient) (2) (*in vivo*) Murine model of *S. suis* meningitis. WT vs. Gb3* deficient mice. *The receptor for *S. suis* **Fhb**.	1-(1) BBB traversal efficiency of *S. suis* was reduced by **Factor H** binding protein (**Fhb**) deficiency and **Fhb** antibody. 1-(2) Gb3 synthesis inhibition reduced adherence of *S. suis* to hCMEC/D3. (2) Gb3 deficiency was protective against meningitis.	**Fhb** facilitates BBB invasion and traversal.	Kong et al., 2017
3	*Streptococcus pneumoniae*	Pneumococcal meningitis (1) (Human) nationwide prospective cohort study of adults with community-acquired bacterial meningitis (n636) vs. control (partners or non-related proxies living in the same dwelling). (2) (*in vivo*) **C5aR** deficient mice.	1-(1) **C5** genotype rs17611 was associated with worse outcome in pneumococcal meningitis patients (OR 2.25; 95% CI 1.33–3.81; *P* = 0.002). Patients with rs17611 GG genotype) had lower CSF WBC counts. 1-(2) **C5a** and **MAC** levels in CSF correlated with admission, death, and worse outcome. 2-(1) CSF WBC count was lower in **C5aR** deficient compared to WT mice. While **C5aR** deficient mice exhibited reduced clinical scores, there was no difference in cerebellar bacterial titers. 2-(2) Anti-**C5** antibody treatment was protective against pneumococcal meningitis.	Complement **C5** activation is associated with poor outcome of pneumococcal meningitis. **C5** inhibition is protective against meningitis.	Woehrl et al., 2011
4	*Streptococcus pneumoniae*	(*in vivo*) Pneumococcal meningitis model using rats infected with *Streptococcus pneumoniae*. Rats treated with **C1** inhibitor (C1-inh.) intravenously or intrathecally.	(1) Intrathecal treatment with **C1**-inh reduced clinical symptoms and inflammatory infiltrate/leukocyte influx. (2) **C1**-inh treatment induced **CR3** expression in the brain.	(a) Inhibition of **classical complement pathway** is protective against bacterial meningitis. (b) **C1**-inh treatment induced upregulation of **CR3**, which may be protective.	Zwijnenburg et al., 2007
5	*Neisseria meningitidis*	(*in vivo*) Human **CD46** transgenic mice vs. WT infected with *N. meningitidis*. (WT and LPS-deficient)	Human **CD46** transgenic mice showed: (1) delayed bacterial clearance from blood and increased serum cytokines (TNF, IL-6, and IL-10). (2) microglial and astroglial activation in the brain. (3) Host immune responses were absent when LPS-deficient meningococci were used.	**CD46** is crucial in the pathogenesis of meningitis.	Johansson et al., 2005
6	*Neisseria meningitidis*	(*in vivo*) Human **CD46** transgenic mice vs. WT infected with *N. meningitidis*.	(1) *N. meningitidis* crossed BBB in **CD46** transgenic but not WT mice. (2) **CD46** transgenic mice had 100% mortality, while 100% of WT mice survived.	Human **CD46** facilitates bacterial translocation across BBB.	Johansson et al., 2003
7	Meningitis-causing bacteria	(Human) Brain tissue autopsy from bacterial meningitis patients	**C3aR** was upregulated and expressed by reactive astrocytes, microglia, and infiltrating macrophages and neutrophils.	**C3aR** is upregulated in glial cells and infiltrating immune cells in the brain.	Gasque et al., 1998
8	*Listeria monocytogenes*	(*in vivo*) Bacterial meningitis model, mice infected with *Listeria monocytogenes*.	**C3** and **FB** mRNA levels were elevated in CSF, neurons and Purkinje cells.	**Alternative complement pathway** (**C3** and **FB**) is activated in the brain.	Stahel et al., 1997

*The relevant complement proteins are highlighted in bold.*

As the long-term sequelae of bacterial meningitis include cognitive impairment, infection-induced inflammation may have lasting or even permanent impact on brain function (van de Beek et al., 2004), and there is strong experimental and clinical evidence for the detrimental role of complement in meningitis. Inhibition of the classical pathway and of C5 were both protective against meningitis caused by *S. pneumoniae* in rodents (Zwijnenburg et al., 2007; Woehrl et al., 2011), and a single nucleotide polymorphism of C5 (rs17611, encoding V802I) was associated with poor outcome in pneumococcal meningitis patients (Woehrl et al., 2011), indicating that the activation of the classical complement pathway plays a key role in the pathogenesis. In murine bacterial meningitis model, *L. monocytogenes* infection caused the up-regulation of C3 and FB mRNA levels in the cerebrospinal fluid (CSF) as well as in parenchymal neurons (Stahel et al., 1997), implicating local activation of the alternative pathway in the brain during meningitis. C3aR is up-regulated in glial cells and infiltrating immune cell populations in the brain tissue autopsy from bacterial meningitis patients (Gasque et al., 1998), and C5aR deficient mice were less susceptible to meningitis development despite unaltered bacterial titers in the brain (Woehrl et al., 2011), suggesting the important roles for anaphylatoxin receptor signaling in infection-induced neuroinflammation.

FH and CD46, the negative regulators of the complement system, contribute to or even enable of bacterial dissemination into the CNS. FH binding protein (Fhb) is a meningococcal protein that binds to human FH. The interaction between Fhb and host FH enables the bacteria to evade the innate immune attack, thereby facilitating systemic dissemination. *Streptococcus suis*, a zoonotic bacterium causing bacterial meningitis (Dominguez-Punaro et al., 2007), expresses Fhb, which contributes to the *S. suis* virulence via inhibition of C3b/iC3b deposition on the bacterial surface (Pian et al., 2012). In addition, Fhb can contribute to the development of severe meningitis by enhancing the traversal of *S. suis* across the BBB, as suggested by *in vivo* and *in vitro* experiments (Kong et al., 2017). Similarly, CD46 acts as the entry receptor for a number of microbes, including *N. meningitidis*, an exclusively human pathogen. While CD46 is ubiquitously expressed in human tissues, it is only expressed in the eye and spermatozoa in mouse (Liszewski et al., 2000; Lyzogubov et al., 2014). *N. meningitidis* does not enter the brain in wild-type mice. However, in genetically engineered mice that express human CD46 (human CD46 transgenic), *N. meningitidis* traversed BBB, leading to significantly higher mortality (Johansson et al., 2003). In addition to delayed bacterial clearance from the circulation, microglial and astroglial activation was observed in the brain of CD46 transgenic mice (Johansson et al., 2005). These data suggest that *N. meningitidis* utilizes CD46 to facilitate its own entry into the CNS.

Systemic infection and sepsis can sensitize the brain to inflammation via complement activation, even without the bacteria entering the CNS. In preterm infants, *S. epidermidis* triggers cerebral inflammation, white matter injury and impaired neurodevelopment (Bi et al., 2015). While Toll-like receptor 2 (TLR2) is thought to be the major contributor to neonatal infection caused by Gram-positive bacteria, the observation that the CSF levels of C1r, C3, and C5 were rapidly increased in *S. epidermidis* infection-induced sepsis model in pigs (Muk et al., 2019) suggests that systemic infection may cause CNS injury via aberrant activation of the complement cascade. Bacterial lipopolysaccharide-induced long-term depression via microglial CR3 activation offers a specific mechanistic link between neuroinflammation, complement activation, synaptic dysfunction, and memory impairments (Zhang et al., 2014).

While complement protects the host against bacterial infection, excessive complement activation can contribute to CNS injury and long lasting or permanent dysfunction. In addition, several complement components and regulators are exploited by bacteria to facilitate their entry into the CNS.

### Viruses

A large number of brain disease-causing viruses, including herpes viruses (human herpes virus 1 [HSV-1], human herpesvirus 6 [HHV-6], and γ-herpesviruses), HIV, measles virus, Borna disease virus (BDV), Theiler’s murine encephalitis virus (TMEV), Venezuelan equine encephalitis virus (VEEV), West Nile virus (WNV), and Zika virus (ZIKV), interact with the complement system in the CNS (Table 1.2) and this interaction may play a role in neurodegeneration. Latent HSV-1 infection is associated with increased risk for AD (Itzhaki, 2018). HHV-6A and HHV-6B are frequently found in patients with neuroinflammatory diseases including MS (Alvarez-Lafuente et al., 2006) and AD (Readhead et al., 2018). γ-herpesviruses, including human Epstein–Barr virus (EBV or HHV-4) and Kaposi’s sarcoma-associated herpesvirus (KSHV or HHV-8), also establish life-long latency in the host, which is associated with increased risk of diverse neurological conditions, including meningitis, encephalitis, HIV-related CNS lymphoma, and MS (Said et al., 1997; Bossolasco et al., 2006; Serafini et al., 2007). Seropositivity for EBV is associated with clinical AD (Carbone et al., 2014). In HIV patients, KSHV infection can manifest in the CNS (Andrews et al., 2011; Baldini et al., 2013; Tso et al., 2017). In addition, HIV can cause cognitive impairment, so called HIV-associated neurocognitive disorders (HAND, NeuroAIDS). Indeed, while antiretroviral therapy effectively inhibits viral replication and reduces mortality, more than 50% of HIV-positive individuals suffer from cognitive impairment (Clifford, 2017; Olivier et al., 2018). Even though antiretroviral therapy suppresses viral replication, neurotoxic HIV proteins, such as Tat, continue to be produced in the CNS, leading to persistent inflammation (Liu et al., 2014; Avalos et al., 2017). WNV is a neuroinvasive pathogen that causes significant neuronal loss, inflammation, and microglial activation (Clarke et al., 2014). WNV targets hippocampal neurons (Armah et al., 2007), and patients recovering from WNV disease often suffer from cognitive impairment (Sejvar et al., 2003; Sadek et al., 2010). ZIKV is the causative agent of Zika fever, congenital infection which leads to severe developmental neurodevelopmental defects, including neonatal microcephaly (Rasmussen et al., 2016; Russo et al., 2017). The fact that ZIKV infection in adults can lead to neurological complications such as acute myelitis, encephalomyelitis, encephalitis, meningoencephalitis, and sensory polyneuropathy (Mécharles et al., 2016; Brito Ferreira et al., 2017), further strengthens the notion that ZIKV is highly neurotropic (Souza et al., 2019). VEEV is endemic to Central and South America and periodically emerges from the enzootic cycle to cause infection in human and equid populations (Weaver and Barrett, 2004), occasionally causing severe encephalomyelitis (Weaver et al., 2004). Measles virus infects over 40 million individuals per year (McChesney and Oldstone, 1989). Acute infection can cause encephalomyelitis, the neurological sequelae of which include subacute sclerosing panencephalitis (SSPE) and measles inclusion body encephalitis (MIBE) (Griffin, 2020).

**TABLE 1.2:** Interactions between complement and viruses in the CNS.

	Pathogen	Study design	Outcomes	Conclusion(s)	References
**Borna disease virus (BDV)**					
1	Borna disease virus (BDV)	(*in vivo*) BDV infection model in rats (intranasal infection)	(1) **C1q** mRNA was upregulated in the brain of BDV-infected rats. (2) **C1q** positive cells (presumably microglia) were preferentially localized in the hippocampus and basolateral cortex.	Local **C1q** expression is induced in the brain after BDV infection.	Dietzschold et al., 1995
**Herpes viruses**					
1	HSV-1	(1) (Human, clinical case report) Herpes simplex virus encephalitis (HSE) in patients. (2) (*in vivo*) HSV-1 infection-induced HSE in **MBL**-deficient mice.	(1) HSE patients had ***MASP2*** heterozygous mutation (G634R and R203W) without defect in TLR3-interferon signaling pathway. Both ***MASP2*** variants induced functional defects in **MASP-2** and reduced antiviral activity. (2) **MBL** deficient mice showed a decreased survival rate and increased HSV-1 burden in the brain.	**The lectin pathway** (**MASP-2** and **MBL**) is involved in the anti-HSV activity. Defect in **the lectin pathway** can lead to higher susceptibility to adult HSE.	Bibert et al., 2019
2	HHV-6A	(*in vitro*) HHV-6A infection model using astroglioblastoma and neuroblastoma cell lines, T cell line, peripheral blood mononuclear cells (PBMC) from healthy donors, and cord blood mononuclear cells (CBMC).	(1) **C3b** induced MSRV-Env expression. (2) Antibodies recognizing SCR3 and/or SCR4 of **CD46** triggered MSRV transactivation. (3) Knockdown of **CD46**-Cyt1 isoform led to loss of HHV-6A-induced MSRV-Env expression.	The engagement of **CD46**-SCR3 and/or **CD46**-SCR4, either by HHV-6A or **C3b**, induces MSRV-Env. HHV-6A-induced MSRV-Env can activate TLR4 signaling.	(Charvet et al., 2018)
3	HSV-1	(*in vitro*) HSV-1 brain infection model using human primary neural cells (neurons + glia).	(1) HSV-1 infection in neural cells caused atrophy and irregular morphology. (2) Infection induced the up-regulation of miRNA-146a and pro-inflammatory mediators, while reducing **FH** protein (known as a miRNA-146a target) levels. ^∗^miRNA-146a: a brain-enriched miRNA	HSV-1 infection in neural cells lead to the down-regulation of **FH** via the induction of miRNA-146a.	(Hill et al., 2009)
4	HHV-6	(*in vitro*) HHV-6 and measles virus (MV) infection-induced fusion model using infected T cells (HSB-2) and primary human glia.	(1) Human oligodendrocytes, astrocytes and microglia express **CD46**. (2) **CD46** inhibition (anti-**CD46** antibody) suppressed the fusion of HHV-6-infected T cells with glial targets (oligodendrocytes and astrocytes).	HHV-6 may spread into the CNS via cell-cell fusion of infected lymphocytes and glial cells, which is mediated by **CD46** on the surface of glia.	Cassiani-Ingoni et al., 2005
5	γ-herpesviruses	(*in vivo*) Murine γ-herpesvirus 68 infection model. Virus: **RCA homolog** (gHB68-RCA) deficient virus vs. WT. Host: **C3** or **FB** deficient vs. WT mice.	(1) gHV68-**RCA** deficient virus exhibited lower infection efficiency in the brain and periphery (lung and spleen) in acute and chronic infection. (2) **C3** deficient caused higher susceptibility to viral latency in the CNS. (3) Lower infection efficiency of gHV68-**RCA** deficient virus compared to WT virus was absent in **C3** deficient mice (but not in **FB** deficient mice).	(a) **C3** prevents viral latency in the CNS. (b) Viral **RCA** protein plays a key role in viral virulence by inhibiting **C3**-dependent host defense.	Kapadia et al., 2002

**RCA: regulators of complement activation**					

	**Study design**		**Outcomes**	**Conclusion**	**References**

**HIV**					
1	(*in vivo*) HAND model: HIV-Tat protein injection into the cerebral cortex in WT and **C1qa** deficient mice.		(1) **C1q** and **C3** were upregulated around injection site and corpus callosum in HAND model (Tat-injected). (2) Iba1^+^microglia increase and synaptic loss were induced in HAND model. (3) **C1q** deficient did not prevent Tat-induced synaptic loss and microgliosis.	(a) Increased **C1q** and **C3**. (b) Microgliosis and synaptic loss induced in HAND are independent of **classical complement cascade (C1q)**. (c) The role of **C3** is unknown.	Hammond et al., 2018
2	(Human) Specimens: CSF and blood from HIV+ subjects with and without cognitive dysfunction. Design: retrospective cross-sectional Populations: HIV+ youth (18–24 years, n20) and older adults (40–46 years, n20) with varying degree of cognitive impairment		(1) CSF **C1q** correlated with NFL in subjects without antiretroviral therapy. (2) A trend towards (*p* = 0.052) elevation of CSF **C1q** expression in subjects with cognitive impairment compared to those with normal cognition.	Increased **C1q** in CSF, which is associated with cognitive impairment.	McGuire et al., 2016
3	(*in vitro*) Recombinant Tat proteins from subtype B HIV-1 isolate (Tat.B) and HIV-1 CRF02_AG (Tat.AG). BBB model: human brain microvascular endothelial cells (HBMEC).		HIV-1 Tat proteins increased the expression of **C3** and **C3b** in HBMEC. Heat inactivated Tat proteins (Tat.B-HI and Tat.AG-HI) also upregulated **C3** and **C3b**.	HIV Tat protein upregulates **C3** and **C3b** in HBMEC, which may cause BBB dysfunction.	(Woollard et al., 2014)
4	(*in vivo*) Neuro-AIDS model using SIV-infected rhesus macaques		(1) **C1q** protein and transcripts colocalized with microglial/macrophage lineage in SIV-encephalitic brains. All SIV-positive cells were also **C1q**-positive. (2) CNS-permeant antiretroviral agent decreased **C1q** synthesis, SIV burden, and inflammation in AIDS-symptomatic macaques.	**Classical complement pathway** activation.	Depboylu et al., 2005
5		(*in vivo*) Neuro-AIDS model using SIV-infected rhesus macaques. Measures: IHC	(1) **C1q** and **C3** levels increased in astrocytes, microglia, and neurons of SIV-infected macaques compared to control. (2) Infiltrating macrophages and multinuclear cells in the brain also expressed **C1q** and **C3** in infected animals. (3) **C1q** and **C3** were deposited on the membrane of neurons.	Viral infection induced **complement activation** (**C1q** and **C3**), which may cause the lysis of bystander neurons.	Speth et al., 2004

**NFL: neurofilament light chain.**					

**Measles virus (MV)**					
1	(*in vitro*) MV infection-induced fusion model using infected T cells (HSB-2) and primary human glia.		1. Human oligodendrocytes, astrocytes and microglia express **CD46**. 2. **CD46** inhibition (anti-**CD46**) suppressed the fusion of MV glycoprotein-expressing T cells with glial targets (oligodendrocytes and astrocytes).	MV may spread into the CNS via cell-cell fusion of infected lymphocytes and glial cells, which is mediated by **CD46** on the surface of glia.	Cassiani-Ingoni et al., 2005
2	(Human) Postmortem subacute sclerosing panencephalitis (SSPE) brain tissue (n4) vs. control with no neurologic disease. Measures: IHC.		1. Strong **CD46** signal was observed on cerebral endothelium throughout the brain as well as on ependymal cells lining the ventricles and choroid plexus, while subsets of neurons and oligodendrocytes were also weakly **CD46** positive. 2. In SSPE brain, cells in MV-infected lesions were negative for **CD46**.	(1) **CD46** is present on endothelial, neuronal, and glial cells in the CNS. (2) MV infection may down-regulate **CD46** in SSPE brain.	McQuaid and Cosby, 2002
3	(*in vivo*) CNS MV infection model using **CD46** transgenic mice^∗^. Intracerebral infection of a vaccine strain of MV. ^∗^Ubiquitously expressing **CD46** with either Cyt1 or Cyt2 cytoplasmic tail.		1. **CD46** transgenic mice (both Cyt1 and Cyt2) were highly susceptible to MV infection compared to non-transgenic control. 2. MV replicated in neurons in the brain. MV induced apoptosis in brain regions, which preceded the death of infected mice.	**CD46** transgenic mice developed progressive infectious measles encephalitis similar to brain disorders in immunocompromised patients.	Evlashev et al., 2000
4	(*in vivo* and *in vitro*) MV infection model using human **CD46** transgenic mice and cells isolated from transgenic mice.		1. **CD46** transgenic mice and murine cells are susceptible to MV infection. 2. MV infection in **CD46** transgenic mice causes the suppression of cellular and humoral immune responses compared to WT. 3. MV infection causes glial activation and T cell infiltration, leading to CNS disease (e.g., seizures) in **CD46** transgenic mice. (4) MV spread throughout CNS via axonal transport.	1. Transgenic mice expressing human **CD46** (on the surface of neurons, lymphocytes, macrophages, and dendritic cells) are susceptible to MV infection and MV-induced CNS disease. 2. MV spread in the CNS via axonal transport.	Oldstone et al., 1999
5	(*in vivo*) MV infection in transgenic mice expressing human CD46 in the CNS (NSE-**CD46**).		1. MV replicates in neurons in the cortex, hippocampus, and thalamus, leading to lethality in CD46 mice. 2. CNS infiltration of T cells, B cells, and macrophages in MV-infected NSE-**CD46** mice, 3. MHC I and II were up-regulated in the CNS, which was associated with astroglial and microglial activation. (4) MV infection induced apoptosis of neurons.	CD46 in the CNS plays a crucial role in MV susceptibility and the development of measles encephalitis.	Manchester et al., 1999
6	(Human) Postmortem brain subacute sclerosing panencephalitis (SSPE) specimens Measures: IHC		**CD46** was down-regulated in heavily infected brain lesions of SSPE, compared to control and SSPE brain regions distant from the lesion.	CD46 expression is reduced by MV infection in SSPE lesions.	Ogata et al., 1997
7	(*in vivo* and *in vitro*) MV infection model in transgenic mice expressing human **CD46** in the CNS (NSE-**CD46**) and primary neurons.		1. MV spread in the brain (hippocampus and cortex) of NSE-**CD46** transgenic mice, but not in WT. 2. MV spread in **CD46** transgenic primary neurons.	**CV46** is essential to CNS infection susceptibility and disease progression.	Rall et al., 1997
**Theiler’s murine encephalomyelitis virus (TMEV)**					
1	(*in vivo*) TMEV-induced seizure model in mice (TMEV intracerebral infection). Measures: FACS. –> Gene expression analysis. Populations: R1 (CD45^low/int^CD11b^+^, ramified microglia) and R2 (CD45^high^CD11b, infiltrating macrophage/activated microglia).		1. R2 population increased from 18 to 72 h after infection. Only R1 was detected in naive. 2. ***C3***, ***C4b*, *C3ar1***, and ***C5ar1*** mRNAs were significantly upregulated. 3. ***C3*** was detected at significantly higher levels in R2 compared to R1 cells. (4) Other immune regulators, such as cytokines and MHC I and II genes, were also upregulated.	(a) TMEV infection induced upregulation of **C3**, **C4b**, **C3aR**, and **C5aR** in the CNS. (b) **C3** was highly expressed in infiltrating macrophages/activated microglia.	Libbey et al., 2017
**Venezuelan equine encephalitis virus (VEEV)**					
1	(*in vivo*) VEEV in infection models in mice (WT vs. **C3** deficient)		(1) VEEV infection via peripheral route caused more severe symptom and enhanced invasion and inflammation in the brain of **C3** deficient mice compared to WT. (2) Direct inoculation of VEEV into the brain led to identical outcomes irrespective of the presence or absence of **C3**.	**C3**-dependent viral clearance in the periphery is critical for the protection against VEEV-induced encephalitis.	Brooke et al., 2012
**West Nile virus (WNV)**					
1	(*in vivo*) Murine WNV disease recovery model using intracranial infection with WNV-NS5-E218A strain. Mouse strains: WT, **C3** deficient and **C3aR** deficient		(1) WNV infection causes synaptic loss in the hippocampal CA3 region. (2) WNV-recovered mice exhibited cognitive deficits without impairment in motor activity. (3) **C1qa** protein colocalized with microglial processes adjacent to neurons. **C3d** colocalized with synaptic terminals. (4) **C3** deficient and **C3aR** deficient mice were protected against WNV-induced synaptic loss.	(a) WNV infection caused complement activation (**C1q**) on microglia and neurons. (b) **C3** and **C3aR** signaling mediate hippocampal synaptic terminal loss in WNV-recovering mice.	Vasek et al., 2016
2	(*in vivo*) Murine WNV infection model using **C3** deficient, **CR1**/**CR2** deficient, and WT (subcutaneous injection).		(1) WNV was detected earlier and in greater levels in the brain and spinal cord of **C3** deficient and **CR1**/**CR2** deficient mice, compared to WT. (2) High levels of WNV antigen were detected primarily in neurons. In WT mice, WNV positive neurons were detected in the cortex, hippocampus, and brain base. Significantly enhanced and more widespread WNV signals were observed throughout the brain in **C3** deficient compared to WT mice. (3) The development of WNV-specific antibodies was blunted in **C3** deficient and **CR1**/**CR2** deficient mice. **C3** deficient and **CR1**/**CR2** deficient mice had higher mortality after exposure to a low dose WNV.	**C3** and **CR1**/**CR2** play a key role in antibody-mediated protection against WNV, thereby reducing the risk of viral dissemination into the brain.	Mehlhop et al., 2005

**Complement receptor 1 (CR1): C3b/C4b receptor, CD35.**					
**Complement receptor 2 (CR2): complement C3d receptor, Epstein-Barr virus receptor, CD21.**					

**Zika virus (ZIKV)**					
	**Study design**		**Outcomes**	**Conclusion**	**References**
1	(1) (*in vivo*) ZIKV infection model in mice (intracerebroventricular infusion). (2) (Human, *ex vivo*) ZIKV infection in human adult cortical tissue *ex vivo*.		(1.1) ZIKV infected mature neurons in the frontal cortex and hippocampus in mice. (1.2) Infection caused microgliosis, the elevation of TNF-a, and upregulation of complement proteins (**C1q** and **C3**). (1.3) Infection caused hippocampal synaptic damage and memory impairment. (2) ZIKV infected mature neurons and replicated in adult human brain tissue.	(a) ZIKV infects neurons in the hippocampus and frontal cortex (b) ZIKV infection causes upregulation of complement proteins (**C1q** and **C3**), which is associated with microglial activation and pro-inflammatory response.	Figueiredo et al., 2019

*The relevant complement proteins are highlighted in bold.*

The complement system provides the first line of defense against viruses, including HSV-1 via the lectin pathway (Bibert et al., 2019), and γ-herpesvirus, VEEV, and WNV via C3-dependent elimination mechanisms (Kapadia et al., 2002; Mehlhop et al., 2005; Brooke et al., 2012). On the other hand, the virus-induced up-regulation of complement components in the CNS may lead to BBB dysfunction and contribute to tissue damage in infection with HIV and TMEV (Speth et al., 2004; Woollard et al., 2014; Libbey et al., 2017). For example, HIV-Tat protein upregulated C3 expression in human brain microvascular endothelial cells (HBMEC) *in vitro* (Woollard et al., 2014), C1q is up-regulated in the brain of Simian immunodeficiency virus (SIV)-infected rhesus macaques (Speth et al., 2004; Depboylu et al., 2005) and mice after HIV-Tat injection into the cerebral cortex (Hammond et al., 2018), two *in vivo* models of NeuroAIDS. Increased C1q in the CSF is associated with cognitive impairment in HIV-infected individuals (McGuire et al., 2016). BDV induced local C1q mRNA expression in the hippocampus and cortex (Dietzschold et al., 1995) and ZIKV infection enhanced expression of C1q and C3, associated with microglial activation and hippocampal synaptic damage and memory impairment in a murine infection model using ZIKV intracerebroventricular infusion (Figueiredo et al., 2019). In murine model induced by intracranial infection of WNV, C1q protein colocalized with microglia adjacent to WNV-infected neurons, possibly causing synaptic terminal loss (Vasek et al., 2016). These data suggest that local complement activation may induce cognitive impairment via the disruption of neurotransmission.

Some viruses increase their virulence by manipulating complement regulatory proteins. HSV-1 infection caused the down-regulation of FH in human primary neuroglia *in vitro* (Hill et al., 2009), potentially leading to enhanced activity of the alternative pathway. As suggested by studies using CD46 transgenic mice, CD46 is involved in the dissemination of measles virus into the CNS, increasing the risk of measles encephalitis (Rall et al., 1997; Manchester et al., 1999; Oldstone et al., 1999; Evlashev et al., 2000). In postmortem SSPE brain tissues, strong CD46 signal was observed on cerebral endothelium throughout the brain, as well as ependymal cells lining the ventricles and choroid plexus (McQuaid and Cosby, 2002), while CD46 expression was suppressed by measles virus infection in SSPE lesions (Ogata et al., 1997; McQuaid and Cosby, 2002). In addition, CD46 expression was observed in primary human glial cells (oligodendrocytes, astrocytes, and microglia) and CD46 inhibition by anti-CD46 antibody suppressed the fusion of glia and measles virus-infected T cells *in vitro*, suggesting that measles virus may spread in the brain parenchyma via binding to CD46 on glia (Cassiani-Ingoni et al., 2005). In HHV-6 infection models *in vitro*, using neuronal and glial cell lines, CD46 also mediated HHV-6 infection-induced transactivation of Multiple Sclerosis-Associated Retrovirus (MSRV) (Charvet et al., 2018). In a murine γ-herpesviruses infection model, C3 deficiency caused higher susceptibility to viral latency in the CNS (Kapadia et al., 2002). Several γ-herpesviruses encode homologues of RCAs (Farriesa and Atkinson, 1991), to inhibit complement activation (Fodor et al., 1995; Kapadia et al., 1999, 2002) thereby preventing complement-mediated elimination (Liszewski et al., 1996; Russo et al., 1996; Virgin et al., 1997). Like bacteria, some viruses can utilize and mimic host RCA proteins for their dissemination and entry into the CNS.

Sustained complement activation is associated with neuroinflammation and neurodegeneration in a number of neurological disorders (Eikelenboom et al., 1989; Rogers et al., 1992). Of note, COVID-19 patients reportedly suffer from diverse neurological complications (Stracciari et al., 2021). Although there is no direct evidence thus far to indicate causality between SARS-CoV-2 infection and neuropathology (Maury et al., 2021), future research will show whether there is a causal link between overactivation of complement in response to SARS-CoV-2 and neurological symptoms or long-term complications of COVID-19 (Garred et al., 2021).

### Fungi

Fungal infection and dissemination into the brain has been proposed to contribute to the etiology of AD (Alonso et al., 2014, 2018). *Candida* is one of the most common commensal fungi, and can cause systemic infections which frequently affect the CNS (Lionakis et al., 2011; Alonso et al., 2014, 2018). Mice with invasive candidiasis exhibit microglial activation and local inflammation (Lionakis et al., 2011). *Aspergillus* and *Cryptococcus* are also fungal species that can cause invasive CNS infection, especially in immunocompromised individuals (Panackal and Williamson, 2015). Similarly, cerebral aspergillosis is caused by *Aspergillus* spp. that mainly affect immunocompromised individuals, such as AIDS patients and those under immunosuppressive treatment regimens (Ruhnke et al., 2007). Cryptococcosis, caused by the encapsulated fungus *Cryptococcus neoformans*, frequently occurs in AIDS, organ transplant recipients and cancer patients, and is the leading cause of mortality in immunocompromised individuals (Rajasingham et al., 2017). These fungal species can also cause meningoencephalitis (Gottfredsson and Perfect, 2000; Góralska et al., 2018), suggesting that fungal infection-induced local inflammation and meningoencephalitis may lead to long-term sequelae including cognitive dysfunction.

The complement system plays pivotal roles in the susceptibility to fungal CNS infection and infection-induced neuropathology (Table 1.3). A study using murine *Candida albican* infection models showed that C5 could be a major determinant of CNS infection susceptibility (Tuite et al., 2005). Neutrophils play a major protective role against dissemination of *C. neoformans* (Lovchik and Lipscomb, 1993; Graybill et al., 1997), and C5aR signaling is required for infection-induced neutrophil recruitment to the brain as suggested by data obtained in a murine infection model using C5 deficiency and inhibitory anti-C5aR antibodies (Sun et al., 2016). C5 and its cleavage product C5a are required for pulmonary accumulation of neutrophils upon infection and intravascular clearance of *C*. *neoformans* (Lovchik and Lipscomb, 1993; Sun et al., 2016), further confirming the crucial roles for C5 and C5a in neutrophil-mediated killing of *C*. *neoformans*. Jointly, these data suggest that complement activation and C5aR signaling are protective against *C. neoformans*-induced meningoencephalitis. In postmortem brain tissues from subjects with cerebral aspergillosis, C1q, C4, C3, and C5 were up-regulated in the brain, and co-localized with neurons, astrocytes, oligodendrocytes, and infiltrating macrophages (Rambach et al., 2008).

**TABLE 1.3:** Interactions between complement and fungi in the CNS.

	Study design	Outcomes	Conclusion	References
***Aspergillus* spp.**				
1	(Human) Postmortem brain tissue specimens from subjects with cerebral aspergillosis (n16): HIV infection (n10), tumor therapy (n2), acute myeloid leukemia (n2), aplastic anemia (n1), organ transplantation (n1). Compared to non-infected control (n7).	(1) **C1q**, **C4**, **C3**, and **C5** were upregulated in the from *Aspergillus*-infected brain. **C1q**, **C4**, **C3**, and **C5** were colocalized with neurons, astrocytes, oligodendrocytes, and infiltrating macrophages. (2) High **complement** levels in the surrounding fibrous layer, but not in the central necrotic area loaded with fungal hyphae.	Complement synthesis increased in astrocytes, neurons, oligodendrocytes, and infiltrating macrophages in the surrounding fibrous layer.	Rambach et al., 2008
***Candida* spp.**				
1	(*in vivo*) Murine model of *Candida albicans* infection. Genetic susceptibility was assessed by intercrossing two inbred strains: A/J (**C5** deficient) and C57BL/6J (B6)	(1) Fungal load in the brain, kidney, and heart was significantly different between the two inbred strains: A/J was more susceptible compared to B6. (2) **C5** was a strong determinant for the brain and kidney susceptibility to infection.	**C5** is a major gene responsible for genetic susceptibility to candidiasis.	Tuite et al., 2005
***Cryptococcus***				
1	(*in vivo*) *Cryptococcus neoformans* infection model in mice (WT, **C3** deficient, and **C5** deficient).	(1) **C3** deficiency, **C5** deficiency, and **C5aR** blockade (specific anti-**C5aR** mAb) reduced the intravascular clearance of *C. neoformans*. (2) Upon infection, neutrophils recruitment to the lung and the brain was less efficient in **C3** deficient and **C5** deficient mice compared to WT. Neutrophil recruitment was also inhibited in WT mice treated with anti-**C5aR**. (3) **C5aR** deficient neutrophils failed to be recruited to the infected lung. (4) Intravascular clearance of disseminating *C. neoformans* is less efficient in the brain compared to the lung. (5) LPS-treatment significantly enhanced neutrophil recruitment to the brain and *C. neoformans* clearance.	(a) Neutrophil recruitment is essential for intravascular clearance of *C. neoformans.* (b) **C5aR**-dependent neutrophil recruitment is crucial for intravascular clearance of *C. neoformans*.	Sun et al., 2016

*The relevant complement proteins are highlighted in bold.*

While the specific mechanistic links between fungal infection and dementia remain to be elucidated (Alonso et al., 2014, 2018), complement may be protective against neurodegenerative disorders via limiting fungal invasion into the CNS.

### Parasites

Protozoan parasites including *Toxoplasma gondii* and *Plasmodium* spp. are obligate intracellular organisms that can potentially cause dysfunction and parenchymal injury in the brain. While *T. gondii* infects all nucleated cells of warm-blooded vertebrates, erythrocytes are the main target in *Plasmodium* infection in vertebrates. Host immune responses to these eukaryotes are very complex (Ivanova et al., 2019). The interactions between the complement system and these protozoa, with relevance for brain pathologies and the potential links to dementia, are summarized in Table 1.4.

**TABLE 1.4:** Interactions between complement and parasites in the CNS.

	Study design	Outcomes	Conclusion	References
***Plasmodium***				
1	(Human) Proteomic analysis on the frontal lobe (autopsy) of subjects with cerebral malaria (CM) caused by *Plasmodium falciparum.*	(1) Proteins associated with innate immune response, complement system (**C1qb**), coagulation, the platelet activation, are elevated in CM. (2) Proteins associated with myelination, oxidative phosphorylation, ROS regulation, sodium and calcium ion transport are depleted in CM.	Innate immune responses (including the **complement** system) and associated demyelination may contribute to the severity of CM.	Kumar et al., 2018
2	(*in vivo*) Experimental Malaria in Pregnancy (EMIP) model using *P. berghei* ANKA in mice (WT vs. **C5aR** deficient).	(1) *In utero* exposure to EMIP induced persistent neurocognitive deficit and affective disorders in the offspring. (2) *In utero* EMIP-induced cognitive deficit in offspring was rescued by genetic or pharmacological disruption of **C5aR** signaling. (3) *In utero* EMIP-induced reduction in neurotransmitter levels (dopamine, 5-HT, and norepinephrine) was observed only in WT (not in **C5aR** deficient) offspring.	*In utero* exposure to MIP induces cognitive deficit in offspring via maternal **C5aR** signaling.	McDonald et al., 2015
3	(*in vivo*) CM model using *P. berghei* ANKA in mice (WT and **C5** deficient).	**C5** deficient mice were protected against infection-induced seizures and high spike frequency.	**C5** plays a role in malaria-induced seizures.	Buckingham et al., 2014
4	(1) (*in vivo*) CM model using *P. berghei* ANKA in mice (WT, **C5aR** deficient, and C5L2 deficient). (2) (Human) Plasma from children presenting with CM or uncomplicated malaria (UM) (case-control)	(1) In experimental CM model, **C5aR** deficient mice (but not C5L2 deficient) showed (moderately) improved survival that was associated with reduced levels of proinflammatory cytokines and chemokine (TNF, IFN-g, and CCL2), as well as preserved endothelial integrity, compared to WT mice. (2) In human subjects, serum **C5a** levels were significantly higher in CM children compared to UM.	Dysregulated **C5aR** signaling contributes to the pathogenesis of CM.	Kim et al., 2014
5	(*in vivo*) CM model using *P. berghei* ANKA in mice (C57BL/6 WT, **C4** deficient, **FB** deficient, and **C3** deficient).	(1) **C4** deficient and **FB** deficient mice were fully susceptible to CM. (2) **C3** deficient mice were partially resistant to CM. (3) Terminal activation (**C5** cleavage) occurred in **C3** deficient mice during CM.	Terminal pathway activation during CM occurs independently of the three upstream pathways, suggesting the crosstalk between coagulation cascade and complement cascade.	Ramos et al., 2012
6	(*in vivo*) CM model in mice using *P. berghei* ANKA. Mouse strains: WT, **C5** deficient, **C5aR** deficient, and **C3aR** deficient.	(1) **C5** deficient mice were resistant to cerebral malaria, whereas **C5aR** deficient and **C3aR** deficient mice were susceptible. (2) **C9** deposition was detected throughout the cortex of infected mice. **C9** deposits frequently colocalized with blood vessels, while some were detected in the parenchyma. (3) anti-**C9** antibody treatment significantly delayed the progress of cerebral malaria.	Protection of **C5** deficient mice against cerebral malaria is mediated through the inhibition of **MAC** formation, not through **C5a**-induced inflammation.	Ramos et al., 2011
7	(*in vivo*) CM model using *P. berghei* ANKA in mice of different genetic backgrounds. ***C5***-deficient: A/J, C57BL/6 with ***C5***-defective allele from A/J, and ***C5***-deficient B10.D2. ***C5***-sufficient: C57BL/6, A/J with C5-sufficient allele from C57BL/6, and *C5*-deficient B10.D2.	(1) CM was associated with the presence of ***C5*** gene. **C5**-sufficient mice were susceptible while and **C5**-deficient mice were CM resistant. (2) **C5a** and **C5aR** blockade rescued susceptible mice from CM.	**C5** and **C5a** are responsible for CM pathogenesis.	Patel et al., 2008
8	(*in vivo*) CM model using *P. berghei* ANKA in mice.	Increased **C1q** and **C5** proteins in the brain of cerebral malaria. **C1q** and **C5** levels correlated with clinical severity.	**C1q** and **C5** are locally upregulated in the brain in cerebral malaria.	Lackner et al., 2008
9	(*in vivo*) CM model using *P. berghei* ANKA in mice (BALB/c; nu/nu and nu/+) (*in vivo*)	(1) Compared to nu/+, nu/nu mice were protected against CM despite higher parasitemia. (2) Early rapid decrease in serum **C3** and increase in serum immune complex levels were observed in nu/+ mice, but not in nu/nu.	T cell-deficiency is protective against CM, which was accompanied by reduced complement activation.	Finley et al., 1982
***Toxoplasma gondii***				
	**Study design**	**Outcomes**	**Conclusion**	**References**
1	(*in vivo* and *in vitro*) *Toxoplasma* infection model: Type II *T. gondii* (Fukaya) in mice (*in vivo*); Type II *T. gondii* (PTG) in murine primary glia (*in vitro*)	(1) mRNA levels of **C1qa**, **C3**, **FB**, **FP**, **C3aR**, and **C5aR** were persistently up-regulated in the infected brain. (2) **C5a** protein was up-regulated in the infected brain. (3) *Toxoplasma* infection in glial cells induced the up-regulation of mRNA for **C1qa**, **FB**, **FP**, and **C5aR** in a microglia-dependent manner.	*Toxoplasma* infection induced the expression of the alternative pathway components (**FB** and **FP**) and anaphylatoxin receptors (**C3aR** and **C5aR**), which was partly mediated by microglia.	Shinjyo et al., 2021
2	(*in vivo*) Chronic *Toxoplasma* infection model: Type II *T. gondii* (Prugniaud) in mice (Kumming). Measures: proteomics using brain tissue samples.	Complement (**C3**, **C4b**, and **C1qa**) and coagulation (e.g., plasminogen) pathways were highly upregulated in the brain of infected mice. Tight junction pathway was disordered.	In *Toxoplasma*-infected brain, complement components (**C3**, **C4b**, and **C1q**) were upregulated possibly causing the disruption of tight junctions.	Huang et al., 2019
3	(*in vivo*) Persistent infection model: Type I *T. gondii* (GT1) in mice (5 months post infection)	(1) Complement **C1q**, **C1r**, **C3**, and **C4** levels were elevated in the brain with high *Toxoplasma* cyst burden. (2) **Complement proteins** were deposited on the surface of degenerating neurons.	*T. gondii* cyst burden is associated with up-regulation of complement components (**C1q**, **C1r**, **C3**, and **C4**), which leads to complement deposition on the surface of degenerating neurons.	Li et al., 2019
4	(*in vivo*) Chronic *Toxoplasma* infection model in mice, using type I (GT1, virulent) and type II (ME49, avirulent) strains.	(1) **C1q** mRNA and protein levels increased after infection. **C1q** levels correlated with *Toxoplasma* cyst burden. (2) **C1q** expression was predominantly cytoplasmic, which was in the cells adjacent to GFAP positive astrocytes, near breached cyst barriers. (3) **C1q** colocalized with *Toxoplasma* cysts in the brain.	*Toxoplasma* infection causes upregulation of **C1q** in the brain, particularly near parasite cysts and punctate synaptic patterns.	Xiao et al., 2016

*The relevant complement proteins are highlighted in bold.*

#### Plasmodium

*Plasmodium* spp. are the agents causing malaria, one of the deadliest infectious diseases worldwide. Cerebral malaria (CM), caused by *P. falciparum*, is the most severe and life-threatening condition characterized by diffuse encephalopathy. CM is frequently accompanied by seizures and coma, and accounts for the majority of childhood deaths from malaria in endemic regions (Schmutzhard and Gerstenbrand, 1984; Idro et al., 2010; Riggle et al., 2020). In addition, more than 10% of children who survive CM have persistent neurological sequelae, including those affecting cognition and behavior (Frevert and Nacer, 2014). Among the features of CM is the adherence of *Plasmodium*-infected red blood cells (iRBCs) to brain vascular endothelium and BBB dysfunction (Yusuf et al., 2017; Pais and Penha-Gonçalves, 2019). Several mechanisms were suggested to explain the severe endothelial damage and vascular leakage in CM, and the proposed hypotheses include hemodynamic hypothesis (Van der Heyde et al., 2006; Wassmer and Grau, 2017), inflammation hypothesis (Silamut et al., 1999; Van der Heyde et al., 2006; Wassmer et al., 2006; Dunst et al., 2017), coagulation dysfunction hypothesis (Grau et al., 2003; Francischetti, 2008; Bridges et al., 2010; OSullivan et al., 2016), and innate immune hypothesis (Pais and Penha-Gonçalves, 2019). However, the exact mechanism remains elusive.

**FIGURE 2:**
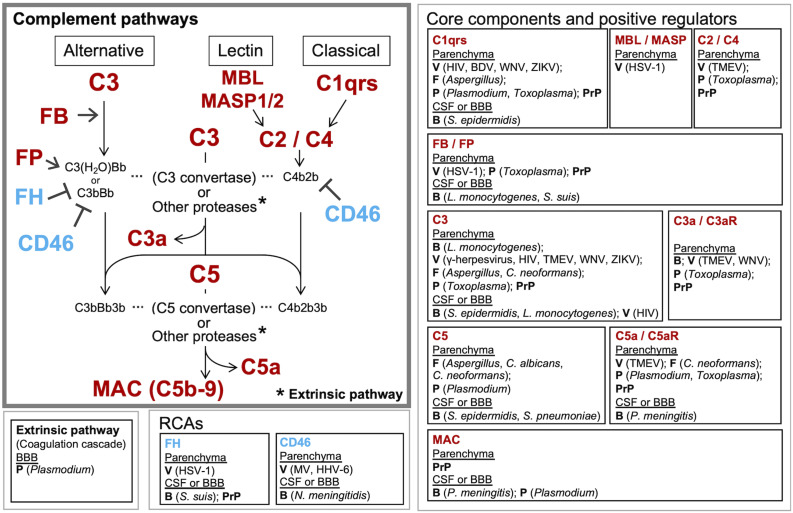
Known and potential interactions between complement proteins and infectious agents. Infectious agents can modulate the expression or activity of complement proteins and receptors in the brain parenchyma, CSF, and BBB. Highlighted are the core complement components/positive regulators (red) and negative regulators (RCAs) (blue), which are potentially affected by infectious agents. Infection-induced activation of the coagulation cascade can also lead to C3 and C5 cleavage via the extrinsic pathway. Pathogens known to interact with the respective complement proteins are presented in the boxes to the right or below the pathway diagram. B, bacteria; V, viruses; F, fungi; P, parasites; PrP, prion proteins.

There is evidence suggesting that the complement system plays a significant role in CM pathogenesis. In an early study using murine CM model with *P. berghei* ANKA (Finley et al., 1982), T cell-deficient (nu/nu) mice were protected against CM compared to nu/+ mice, despite comparable parasitemia. The rapid decrease in serum C3 and concomitant increase in serum immune complexes observed in wildtype mice were absent in nu/nu mice, indicating the interaction between the complement system and cellular immunity in CM (Finley et al., 1982). C1q and C5 protein levels were also significantly higher in the brain of CM mice compared to non-CM mice, suggesting the activation of the classical pathway (Lackner et al., 2008). The roles for the complement system in CM were further examined using genetically modified mice, including mice deficient in C5 (Patel et al., 2008; Ramos et al., 2011; Buckingham et al., 2014), C5aR (Ramos et al., 2011; Kim et al., 2014; McDonald et al., 2015), C3 (Ramos et al., 2012), C4 (Ramos et al., 2012), FB (Ramos et al., 2012), and C3aR (Ramos et al., 2011). Notably, C5 deficient mice were fully protected from CM and CM-associated seizures (Ramos et al., 2011; Buckingham et al., 2014), while C5aR deficient mice were only moderately protected (Ramos et al., 2011; Kim et al., 2014). Mice lacking FB, C4, or C3aR showed outcomes comparable to wildtype mice (Ramos et al., 2011, 2012). In addition, C9 deposits were observed in the CNS during CM, and C9 inhibition (neutralizing anti-C9 antibody injection) significantly delayed CM development (Ramos et al., 2011). Results from clinical studies also show complement activation during CM. Serum C5a levels were significantly higher in children with CM compared to those without CM (Kim et al., 2014). Postmortem CM frontal lobes exhibited activation of complement, the coagulation cascade, and platelets (Kumar et al., 2018). These data suggest that MAC formation plays a critical role in CM pathogenesis. In addition, persistent cognitive deficits caused in the offspring by *in utero* exposure to malaria were dependent on maternal C5a-C5aR signaling (McDonald et al., 2015). These findings point to the role of C5a in the initiation of neuroinflammation together with the dysregulation of angiogenesis and synaptogenesis (McDonald et al., 2013). Surprisingly, the formation of conventional C5 convertase via C3 activation was not required for CM progression (Ramos et al., 2012), which suggests that C5 was activated by coagulation factors or other non-complement proteases (Ramos et al., 2012; Figure 2). These observations support the role for the complement system as a link between immune responses and dysregulated coagulation during CM.

The interactions between *Plasmodium* and the complement system are possibly even more intricate (Schmidt et al., 2015; Kennedy et al., 2016, 2017; Rosa et al., 2016). Blood stage *P. falciparum* (free merozoites as well as intraerythrocytic schizonts) evades complement-mediated destruction by recruiting FH, a major negative regulator of complement activation, on the cell surface (Kennedy et al., 2016; Rosa et al., 2016). As mentioned above, several other pathogens, including *Neisseria meningitidis* (Granoff et al., 2009; Schneider et al., 2009) and *Borrelia burgdorferi* (Kraiczy et al., 2001), use FH for the same purpose, suggesting that it is an evolutionarily conserved strategy to circumvent complement-mediated elimination. *Plasmodium* utilizes another complement regulatory protein CR1 as a receptor for red blood cell invasion (Tham et al., 2010; Spadafora et al., 2010). In addition, mobilization of CR1 on the surface of *Plasmodium*-infected red blood cells is required for rosetting (Rowe et al., 1997), a process implicated in vascular obstruction during severe malaria (Carlson, 1993). CR1 polymorphisms could explain the association between altered efficiency of rosetting and malaria severity (Cockburn et al., 2004; Schmidt et al., 2015).

In conclusion, overactivation of complement cascade triggered by the parasite conceivably contributes to CM pathogenesis, and the inhibition of the complement cascade at the level or downstream of C5 activation, and/or the inhibition of CR1 could be a beneficial treatment strategy for CM.

#### Toxoplasma gondii

*Toxoplasma gondii* infects approximately one-third of the world population. It is an opportunistic infection that in individuals with immunodeficiency can cause severe diseases, including *Toxoplasma* encephalitis (Marra, 2018). Infection during pregnancy can lead to congenital toxoplasmosis, the severity of which depends on the stage of pregnancy (Bigna et al., 2020). While *Toxoplasma* rarely causes symptoms in healthy individuals with effective immunity, it can establish latent infection in the brain and other tissues (Pittman and Knoll, 2015). It is also notable that the virulence can vary depending on *Toxoplasma* genotype (Howe and Sibley, 1995; Behnke et al., 2011; Pomares et al., 2018; Taniguchi et al., 2018). Once disseminated into the brain, the parasite transforms into bradyzoite form (cyst) and establishes a life-long infection in neurons and glia (Ólafsson and Barragan, 2020). The presence of *Toxoplasma* cysts in the CNS has been linked to various neuropsychiatric disorders, such as schizophrenia (Elsheikha et al., 2016; Del Grande et al., 2017; Fuglewicz et al., 2017; Fond et al., 2018; Stepanova et al., 2019; Tyebji et al., 2019a, b), possibly via the direct modulation of dopaminergic/serotonergic signaling as well as neuroinflammation (Henriquez et al., 2009; Mahmoud et al., 2017). T cells and type II interferon (IFN-γ)-dependent immune responses are an essential part of the host defense against *Toxoplasma* infection (Sasai et al., 2018). Type I interferons (IFNs) also play an important regulatory role in the innate immune response during protozoan infection, including toxoplasmosis (Silva-Barrios and Stäger, 2017).

*Toxoplasma* is classified into three groups: virulent type I, which causes acute infection, and avirulent type II and type III, which are responsible for chronic infection. Several studies have suggested the involvement of the complement system during acute *Toxoplasma* (type I) infection. *Toxoplasma* lytic activity of human sera was dependent on classical pathway components (C1q, C2, C4, C5, C6, C7, and C8) in a study using a virulent *Toxoplasma* strain *in vitro* (Schreiber and Feldman, 1980). Animal sera (pig, rabbit, and dog), with the exception of cat serum, effectively killed *Toxoplasma* tachyzoites of a virulent strain via C1q and natural IgM-dependent complement activation (Kaneko et al., 2004). *Toxoplasma* tachyzoites of the same strain were resistant to complement-mediated lysis via the formation of iC3b, the inactive form of C3b (Fuhrman and Joiner, 1989). *Toxoplasma* tachyzoites of both virulent and avirulent strains mobilized complement regulatory proteins (FH and C4BP) on their surface to evade complement-mediated parasite killing *in vitro*, whereas C3 deficient mice suffered higher parasite burden in the brain and other organs *in vivo*, suggesting complex host-parasite interactions (Sikorski et al., 2020). Of note, C5 deficiency rendered either protection or enhanced mortality depending on the genetic background of the murine host (Araujo et al., 1975). These data suggest that, while the complement system plays a pivotal role in the protection against acute *Toxoplasma* infection, the parasite has evolved strategies to manipulate the complement system to survive and propagate in the host.

The interaction between *Toxoplasma* and the complement system in the brain has recently been elucidated. In murine *Toxoplasma* infection models, cerebral C1q is upregulated during chronic infection with virulent and avirulent strains (Xiao et al., 2016), and persistent infection with a virulent *Toxoplasma* strain led to the upregulation of C1q, C1r, C3, and C4 mRNA levels and deposition of complement component proteins (C1q and C3) in the brain. These changes were associated with neurodegeneration (Li et al., 2019). An avirulent *Toxoplasma* strain also caused upregulation of complement proteins, including C3, C4b, and C1q, in the mouse brain (Huang et al., 2019). Furthermore, mRNAs for FB and FP, C3aR, and C5aR were up-regulated in the brain of mice chronically infected with an avirulent *Toxoplasma* strain (Shinjyo et al., 2021), suggesting that the alternative pathway is activated in the brain during chronic infection. The *Toxoplasma* infection-induced up-regulation of FB, FP, and C5aR mRNAs occurred in primary murine glial cells *in vitro* in a microglia-dependent manner (Shinjyo et al., 2021). These data suggest that, while complement-dependent clearance is essential in the initial, peripheral phase of infection, chronic *Toxoplasma* infection can cause persistent complement activation in the CNS.

### Prion Proteins

Transmissible spongiform encephalopathies (TSEs, prion diseases) are fatal neurodegenerative diseases, which include bovine spongiform encephalopathy (BSE) in cattle, scrapie in sheep and goat, Creutzfeldt-Jakob disease (CJD), fatal familial insomnia, and Gerstmann-Sträussler-Scheinker syndrome in humans (Prusiner et al., 1998; Prusiner, 1998). Most neurodegenerative diseases, including prion diseases and AD, share two common features: the accumulation and self-propagation of misfolded proteins (Gomez-Gutierrez and Morales, 2020). TSE-causing infectious prion proteins (PrP^Sc^) transmit the disease-associated conformation to normal prion proteins (PrP^C^), leading to further propagation of PrP^Sc^. Following peripheral exposure and prior to neuroinvasion, PrP^Sc^ accumulates in lymphoid tissues, including lymph nodes and Peyer’s patches. Early PrP^Sc^ accumulation occurs within the germinal center on follicular dendritic cells (FDCs) (Brown et al., 1999) as well as within tingible body macrophages (McGovern and Jeffrey, 2007). From the lymphoid tissues, transmission to the CNS occurs via the peripheral nervous system (Glatzel et al., 2001). The central event in prion disease progression is the accumulation of PrP^Sc^ in the CNS accompanied by neuronal loss and spongiform neuropils, suggesting aggressive destruction of neural networks (Budka, 2000).

The involvement of the complement system in prion-induced neuropathology has been observed in different types of prion diseases (Bonifati and Kishore, 2007; Table 1.5). In scrapie-infected rodents, total complement activity in the brain was significantly increased at the terminal stage (Lv et al., 2014). In addition, C1q upregulation (Dandoy-Dron et al., 1998, 2000; Carroll et al., 2018), C3 upregulation and colocalization with neurons, and MAC deposition on neurons were observed in infected human brain (Kovacs et al., 2004) as well as in scrapie model in mice (Lv et al., 2014), suggesting the activation of the classical and terminal pathways. C1q also colocalized with infected neurons *in vitro*, and throughout the brain C1q distribution overlapped with PrP (Hasebe et al., 2012). In addition, FB and FP levels significantly increased in the brain of scrapie-infected mice, while FB and C3 colocalized with neurons and activated microglia (Chen et al., 2020), suggesting the activation of the alternative pathway and microglia. Notably, mice deficient in C1q or FB/C2 were protected against encephalopathy after intraperitoneal prion exposure, but C3 deficiency was not protective (Klein et al., 2001). Interestingly, reactive astrocytes in prion diseases are characterized by C3 up-regulation and mixed A1/A2 phenotype (Hartmann et al., 2019) distinct from neurotoxic astrocytes observed in other neurodegenerative diseases (Kwon and Koh, 2020). The abolishment of C3-positive astrocytes led to prion disease acceleration (Hartmann et al., 2019). These data suggest that C3 plays multiple roles in prion disease progression, depending on the context. In contrast, complement hemolytic activity was significantly lower in the CSF of CJD patients, particularly in genetic CJD cases (Chen et al., 2016). In addition, the protein levels of some complement components, including C3, C4, and C9, decreased in the CSF of sporadic CJD (sCJD) patients compared to non-CJD group (Chen et al., 2016). While the explanation conceivably lies in the increased consumption of complement in the brain parenchyma, the neuropathologic implications of these findings are currently elusive.

**TABLE 1.5:** Interactions between complement and prion proteins in the CNS.

	Pathogen (disease)	Study design	Outcomes	Conclusion	References
1	Scrapie (Prion disease)	(*in vivo*) Prion disease model: scrapie-infected mice	(1) **FB** and **FP** (properdin) levels significantly increased in the brain of scrapie-infected mice. (2) **FB** and **C3** colocalization was observed with neurons and activated microglia, but not with astrocytes.	**The alternative pathway** is activated and plays a role in triggering the complement cascade in the brain during prion infection-induced neuropathogenesis.	Chen et al., 2020
2	Human sCJD and scrapie	(1) (Human) Postmortem sCJD brain specimens (2) (*in vivo*) Prion disease model in mice (WT vs. Triple deficient lacking TNF-α, IL-1α and **C1qa)**	(1) **C3**^+^-astrocytes (A1-like-astrocytes) were abundant in mouse and human prion diseases. (2) Mice lacking TNF-α, IL-1α and **C1qa** had accelerated prion disease course (measured by survival rate) without affecting the formation of PrP^Sc^ and microglial activation. (3) ***C3*** expression was significantly up-regulated and **C3**^+^-astrocytes significantly increased in the thalamus of terminally sick WT but not in Triple deficient mice.	Reactive astrocyte signature in prion diseases is characterized by upregulation of **C3** and a mixed A1/A2 phenotype, which is distinct from other neurodegenerative diseases. **C3** expression in astrocytes may be protective in prion disease.	Hartmann et al., 2019
3	Scrapie (Prion disease)	(*in vivo*) Prion disease model: scrapie infection in mice (WT, TLR2 deficient, **C3aR** deficient, and **C5aR** deficient).	(1) RNA levels of complement components (***C4b***, ***C1qa***, ***C1qb***, and ***C1qc***), DAMP receptors (i.e., *Tlr2*, *Tlr4*, *Tlr8*, ***C3ar1*** and ***C5ar1***) were upregulated in the thalamus and the whole brain. (2) TLR2 deficient, but not **C3aR** deficient and **C5aR** deficient, caused higher susceptibility to prion disease. (3) TLR2 deficient or **C5aR** deficient did not alter the transcription of proinflammatory genes.	(a) Scrapie infection induced enhanced expression of complement components (**C4b** and **C1q**) as well as anaphylatoxin receptors (**C3aR** and **C5aR**). (b) **C3aR** or **C5aR** signaling does not play a major role in prion diseases.	Carroll et al., 2018
4	Scrapie (Prion disease)	(*in vivo*) **FH** deficient mouse prion disease model (scrapie) using transgenic mice expressing zero (**FH**–/–), one (**FH**+/–), or two (**FH**+/+) allelic copies of ***Cfh***.	(1) Brain PrP loads correlated with ***Cfh*** expression. (2) Splenic propagation and clinical manifestation were delayed by **FH** deficiency. (3) **FH** directly interacts with PrP^Sc^.	(a) **FH** enhances scrapie-induced brain prion load. (b) **FH** directly binds prions.	Kane et al., 2017
5	Human sCJD	(Human) CSF from prion disease patients and non-prion disease patients.	(1) **Complement** hemolytic activity (CH50) was lower in the CSF from sCJD patients. CH50 was consistently lower in genetic prion disease patients. (2) **C3**, **C4**, and **C9** protein levels were lower in the CSF from sCJD patients.	**Complement** activity is down-regulated in the CSF of CJD patients, conceivably through consumption.	Chen et al., 2016
6	Scrapie (prion disease)	(*in vivo*) Prion disease model (scrapie) in mice and hamster: 139A-infected mice and 263K-infected hamster	(1) Total **complement activity** (CH50) was higher in the brain of scrapie-infected rodents. (2) **C1q** was upregulated in the brain of scrapie-infected rodents. (3) Stronger **C3** signals in scrapie-infected brain. **C3** colocalized with astrocytes, microglia, and neurons. (4) **MAC** was deposited in the infected brain and colocalized with neurons.	The activation of the complement system may be a hallmark during prion infection.	Lv et al., 2014
7	Scrapie (prion disease)	(*in vivo* and *in vitro*) Scrapie (Chandler and 22L) infection model: mice and N2a cells.	(1) **C1q** colocalized with PrP in Chandler-infected N2a cells, while **C3** colocalized with PrP in 22L-infected N2a cells. (2) **C1q** colocalized with PrP throughout the brain and mild **C3** deposition was detected in the cerebral cortex, septum, thalamus, midbrain, and pons in Chandler-infected brain. **C1q** was absent in the dorsal part of thalamus and **C3** was more pronounced in the thalamus compared to Chandler-infected brain.	Prion-induced complement activation is PrP strain dependent.	Hasebe et al., 2012

*The relevant complement proteins are highlighted in bold.*

Given that FH deficiency was protective against PrP propagation (Kane et al., 2017), prion proteins may escape complement-mediated clearance by manipulating FH-dependent complement inhibition, possibly via direct interaction between PrP^Sc^ and FH (Kane et al., 2017). Thus, RCAs seem important target molecules exploited by infectious agents, including prion proteins, to evade innate immunity and propagate throughout the body including the CNS.

### Complement: A Double Edge Sword in the Infected Brain

The consequences of local complement activation by infectious agents for brain tissue integrity and function are highly context dependent. Complement has a role in the regulation of normal neuronal functioning and homeostasis. It may be neuroprotective and play a critical role in the elimination of various pathogens, but can also be a major mediator of neurodegeneration and tissue damage. The complement system can also be hijacked by some pathogens and play a complicit role in their dissemination. Figure 2 illustrates the potential interactions between the complement system and infectious agents, and Table 2 summarizes the responses of selected complement proteins to the individual pathogens, including up- or down-regulation, deposition, and the impact of genetic polymorphism or genetic manipulations.

**TABLE 2:** The functions of specific complement proteins and in CNS infection.

	Pathogen	Observation	Function	References
**Classical pathway**				
Bacteria				
1	*Staphylococcus epidermidis*	C1r is up-regulated in CSF.	Unknown	Muk et al., 2019
2	*Streptococcus pneumoniae*	C1 inhibitor is protective against meningitis.	Harmful	Zwijnenburg et al., 2007
Viruses				
1	BDV	C1q is up-regulated in the brain.	Unknown	Dietzschold et al., 1995
2	HIV (Tat protein)	C1q is up-regulated in the brain (corpus callosum), however, C1q deficiency does not prevent HIV-induced synaptic loss and microgliosis.	Not harmful	Hammond et al., 2018
3	HIV	CSF C1q is associated with cognitive impairment.	Potentially harmful	McGuire et al., 2016
4	HIV	C1q is up-regulated and colocalizes with microglia/macrophages in the infected brain.	Unknown	Depboylu et al., 2005
5	HIV	C1q is up-regulated in astrocytes, microglia, and neuron, expressed in infiltrating immune cells, and deposited on the membrane of neurons.	Unknown	Speth et al., 2004
6	WNV	C1q deposition on microglial processes adjacent to WNV-positive neurons, which may facilitate synaptic loss.	Unknown	Vasek et al., 2016
7	ZIKV	C1q is up-regulated in the CNS, which is associated with microglial activation.	Unknown	Figueiredo et al., 2019
Fungi				
1	*Aspergillus* spp.	C1q is up-regulated in the postmortem brain from cerebral aspergillosis subjects. Complement proteins co-localized with neurons, astrocytes, oligodendrocytes, and infiltrating macrophages. Higher levels in the surrounding fibrous layer.	Unknown	Rambach et al., 2008
Parasites				
1	*Plasmodium falciparum*	C1q is up-regulated in the frontal lobe of cerebral malaria patients.	Unknown	Kumar et al., 2018
2	*Plasmodium* spp.	C1q is up-regulated in the brain of cerebral malaria, correlation with clinical severity.	Potentially harmful	Lackner et al., 2008
3	*Toxoplasma gondii*	C1q is up-regulated in the cerebral cortex and glial cells.	Unknown	Shinjyo et al., 2021
4	*Toxoplasma gondii*	C1q is up-regulated in the brain.	Unknown	Huang et al., 2019
5	*Toxoplasma gondii*	C1q and C1r are up-regulated in the brain with high cyst burden.	Unknown	Li et al., 2019
6	*Toxoplasma gondii*	C1q is up-regulated in the brain.	Unknown	Xiao et al., 2016
Prion proteins				
1	Prion (scrapie)	C1q is up-regulated in the brain.	Unknown	Carroll et al., 2018
2	Prion (scrapie)	C1q is up-regulated in the brain.	Unknown	Lv et al., 2014
3	Prion (scrapie)	C1q and complement receptor mediate PrP^Sc^ uptake by cDCs in the periphery	Potentially beneficial in the initial phase.	Flores-Langarica et al., 2009
4	Prion (scrapie)	C1q colocalized with PrP in neurons.	Unknown	Kovacs et al., 2004
5	Prion (scrapie)	C1q deficiency is protective against hippocampal neuropathogenesis.	Harmful	Klein et al., 2001
6	Prion (BSE and scrapie)	C1q is up-regulated in the brain.	Unknown	Dandoy-Dron et al., 2000
7	Prion (scrapie)	C1q is up-regulated in the brain.	Unknown	Dandoy-Dron et al., 1998
**Lectin pathway**				
Viruses				
1	HSV-1	Defects in the lectin pathway (MBL and MASP) can cause higher viral burden in the brain and susceptibility to adult Herpes simplex virus encephalitis.	Beneficial	Bibert et al., 2019
**Classical pathway or Lectin pathway**				
Viruses				
1	TMEV	C4b is up-regulated in the CNS.	Unknown	Libbey et al., 2017
Parasites				
1	*Toxoplasma gondii*	C4b is up-regulated in the brain.	Unknown	Huang et al., 2019
2	*Toxoplasma gondii*	C4 is up-regulated in the brain with high cyst burden.	Unknown	Li et al., 2019
Proteins				
1	Prion (scrapie)	C4b is up-regulated in the brain.	Unknown	Carroll et al., 2018
2	Prion (scrapie)	C2 contributes to hippocampal neuropathogenesis.	Harmful	Klein et al., 2001
**Alternative pathway**				
Bacteria				
1	*Listeria monocytogenes*	FB is up-regulated in CSF and neurons.	Unknown	Stahel et al., 1997
2	*Streptococcus suis*	Bacterial Factor H binding protein (Fhb) facilitates BBB traversal.	Potentially complicit	Kong et al., 2017
Parasites				
1	*Toxoplasma gondii*	FB and FP are up-regulated in the cerebral cortex and glial cells.	Unknown	Shinjyo et al., 2021
Proteins				
1	Prion (scrapie)	FB and FP are up-regulated in the brain.	Unknown	Chen et al., 2020
2	Prion (scrapie)	FB facilitates hippocampal neuropathogenesis.	Harmful	Klein et al., 2001
**C3**				
Bacteria				
1	*Staphylococcus epidermidis*	C3 is up-regulated in CSF.	Unknown	Muk et al., 2019
2	*Listeria monocytogenes*	C3 is up-regulated in CSF and neurons.	Unknown	Stahel et al., 1997
Viruses				
1	γ-herpesvirus	C3 prevents viral latency in the CNS.	Beneficial	Kapadia et al., 2002
2	HIV	C3 is up-regulated in the brain (corpus callosum).	Unknown	Hammond et al., 2018
3	HIV (Tat protein)	C3 and C3b are up-regulated in BBB endothelial cells.	Unknown	Woollard et al., 2014
4	HIV	C1q is up-regulated in astrocytes, microglia, and neuron, expressed in infiltrating immune cells, and deposited on the membrane of neurons.	Unknown	Speth et al., 2004
5	TMEV	C3 is up-regulated in the CNS.	Unknown	Libbey et al., 2017
6	VEEV	C3-dependent viral clearance in the periphery is protective against VEEV-induced encephalitis.	Beneficial	Brooke et al., 2012
7	WNV	C3d deposition on synaptic terminals, which may facilitate synaptic loss.	Potentially harmful	Vasek et al., 2016
8	WNV	C3 is protective against WNV dissemination in the CNS. C3 plays a role in WNV-specific antibody development.	Beneficial	Mehlhop et al., 2005
9	ZIKV	C3 is up-regulated in the CNS, association with microglial activation.	Unknown	Figueiredo et al., 2019
Fungi				
1	*Aspergillus* spp.	C3 is up-regulated in the postmortem brain from cerebral aspergillosis subjects. Co-localized with neurons, astrocytes, oligodendrocytes, and infiltrating macrophages. Higher levels in the surrounding fibrous layer.	Unknown	Rambach et al., 2008
2	*Cryptococcus neoformans*	C3 facilitates the intravascular clearance of *C. neoformans*.	Beneficial	Sun et al., 2016
Parasites				
1	*Toxoplasma gondii*	C3 is up-regulated in the brain.	Unknown	Huang et al., 2019
2	*Toxoplasma gondii*	C3 is up-regulated in the cerebral cortex and glial cells.	Unknown	Shinjyo et al., 2021
3	*Toxoplasma gondii*	C3 is up-regulated in the brain with high cyst burden.	Unknown	Li et al., 2019
Proteins				
1	Prion (scrapie and human sCJD)	C3 is up-regulated in A1/A2-mixed astrocytes in the brain.	Unknown	Hartmann et al., 2019
2	Prion (scrapie)	C3 is up-regulated and colocalized with neurons and glia in the brain.	Unknown	Lv et al., 2014
3	Prion (scrapie)	C3b colocalized with PrP in neurons.	Unknown	Kovacs et al., 2004
4	Prion (scrapie)	C3 deficiency is NOT protective against prion-induced hippocampal neuropathogenesis.	Not harmful	Klein et al., 2001
**C5**				
Bacteria				
1	*Staphylococcus epidermidis*	C5 up-regulation in CSF.	Unknown	Muk et al., 2019
2	*Streptococcus pneumoniae*	C5 inhibition is protective against meningitis.	Potentially harmful	Woehrl et al., 2011
Fungi				
1	*Aspergillus* spp.	C3 is up-regulated in the postmortem brain from cerebral aspergillosis subjects. Complement co-localized with neurons, astrocytes, oligodendrocytes, and infiltrating macrophages. Higher levels in the surrounding fibrous layer.	Unknown	Rambach et al., 2008
2	*Candida albicans*	C5 deficiency is associated with fungal dissemination, including in the brain.	Beneficial	Tuite et al., 2005
3	*Cryptococcus neoformans*	C5 facilitates the intravascular clearance of *C. neoformans*.	Beneficial	Sun et al., 2016
Parasites				
1	*Plasmodium* spp.	C5 deficiency is protective against infection-induced seizures.	Harmful	Buckingham et al., 2014
2	*Plasmodium* spp.	C5 deficiency is protective against cerebral malaria development.	Harmful	Ramos et al., 2011
3	*Plasmodium* spp.	C5 deficiency is protective against cerebral malaria development.	Harmful	Patel et al., 2008
4	*Plasmodium* spp.	C5 is up-regulated in the brain of cerebral malaria, correlates with clinical severity.	Unknown	Lackner et al., 2008
5	*Taenia solium*	C5 polymorphism is associated with the risk of developing neurocysticercosis.	Unknown	Villegas et al., 2019
**MAC**				
Parasites				
1	*Plasmodium* spp.	C9 deposits throughout the cortex of cerebral malaria, frequently colocalizing with blood vessels. Anti-C9 antibody treatment delayed the progress of cerebral malaria.	Harmful	Ramos et al., 2011
2	Prion (scrapie)	MAC deposits and colocalized with neurons in the brain.	Unknown	Lv et al., 2014
3	Prion (scrapie)	MAC formation is correlated with severity of neuropathology.	Potentially harmful	Kovacs et al., 2004
**C3aR**				
Bacteria				
1	Meningitis causing bacteria	Up-regulated in reactive astrocytes, microglia, and infiltrating immune cells.	Unknown	Gasque et al., 1998
Viruses				
1	TMEV	C3aR is up-regulated in the CNS.	Unknown	Libbey et al., 2017
2	WNV	C3aR deficiency is protective against WNV-induced synaptic loss.	Harmful	Vasek et al., 2016
Parasites				
1	*Toxoplasma gondii*	C3aR is up-regulated in the cerebral cortex.	Unknown	Shinjyo et al., 2021
Proteins				
2	Prion (scrapie)	C3aR is up-regulated in the brain. C3aR deficiency was not protective against neuropathology.	Not harmful	Carroll et al., 2018
**C5aR**				
Bacteria				
1	*Streptococcus pneumoniae*	C5aR deficiency is protective against meningitis, while conferring no impact on bacterial titer.	Potentially harmful	Woehrl et al., 2011
Viruses				
1	TMEV	C5aR is up-regulated in the CNS.	Unknown	Libbey et al., 2017
Fungi				
1	*Cryptococcus neoformans*	C5aR-dependent neutrophil recruitment mediates intravascular clearance of *C. neoformans*.	Beneficial	Sun et al., 2016
Parasites				
1	*Plasmodium* spp.	C5aR mediates in utero malaria exposure-induced persistent neurocognitive deficits.	Harmful	McDonald et al., 2015
2	*Plasmodium* spp.	C5a is up-regulated C5aR deficiency was protective against in cerebral malaria.	Harmful	Kim et al., 2014
3	*Plasmodium* spp.	C5aR blockade was protective against cerebral malaria.	Harmful	Patel et al., 2008
4	*Toxoplasma gondii*	C5aR is up-regulated in the cerebral cortex and glial cells.	Unknown	Shinjyo et al., 2021
Proteins				
1	Prion (scrapie)	C5aR is up-regulated in the brain. C5aR deficiency was not protective against neuropathology.	Not harmful	Carroll et al., 2018
**Regulators of complement activation (RCAs)**				
**CR1/CR2 (CD35/CD21)**				
Viruses				
1	West Nile virus (WNV)	CR1/CR2 is protective against WNV dissemination in the CNS. CR1/CR2 plays a role in WNV-specific antibody development.	Beneficial	Mehlhop et al., 2005
**CD46**				
Bacteria				
1	*Neisseria meningitidis*	Human CD46 mediates meningitis development.	Complicit	Johansson et al., 2005
2	*Neisseria meningitidis*	Human CD46 mediates BBB traversal.	Complicit	Johansson et al., 2003
Viruses				
1	HHV-6A	Human CD46 mediates HHV-6A induced transactivation of MSRV-Env, which leads to TLR4 activation.	Unknown	Charvet et al., 2018
2	HHV-6	Human CD46 facilitates viral dissemination into the CNS via mediating cell-cell fusion of infected lymphocytes and glial cells.	Complicit	Cassiani-Ingoni et al., 2005
3	Measles virus	Human CD46 is down-regulated in the postmortem brain of Measles virus-induced subacute sclerosing panencephalitis subjects.	Unknown	McQuaid and Cosby, 2002
4	Measles virus	Human CD46 mediates progressive viral dissemination and the development of encephalitis.	Complicit	Evlashev et al., 2000
5	Measles virus	Human CD46 causes the suppression of immune responses caused by viral infection, and facilitates viral dissemination into the CNS, leading to glial activation and T cell infiltration.	Complicit Eventually harmful	Oldstone et al., 1999
6	Measles virus	Human CD46 expression in neurons facilitates viral replication in the brain, and causes the infiltration of immune cells into the CNS and lethality.	Complicit Eventually harmful	Manchester et al., 1999
7	Measles virus	CD46 is down-regulated in the heavily infected brain lesions of SSPE patients.	Unknown	Ogata et al., 1997
8	Measles virus	Human CD46 expression in neurons facilitates viral dissemination in the brain (hippocampus and cortex).	Complicit	Rall et al., 1997
**FH**				
Bacteria				
1	*Streptococcus suis*	Bacterial Factor H binding protein (Fhb) facilitates BBB traversal.	Potentially complicit	Kong et al., 2017
Viruses				
1	HSV-1	FH is down-regulated in infected neural cells.	Unknown	Hill et al., 2009
Proteins				
1	Prion (scrapie)	FH directly interacts with PrP^Sc^. FH facilitates disease propagation.	Potentially complicit	Kane et al., 2017
**RCA homolog**				
Viruses				
1	γ-herpesvirus	Viral RCA homologue increases viral virulence via inhibiting C3-dependent host defense.	Manipulated	Kapadia et al., 2002

*The relevant complement proteins are highlighted in bold.*

In the CNS, C1q plays potentially harmful roles in bacterial (*S. pneumoniae*-induced bacterial meningitis) (Zwijnenburg et al., 2007), viral (HIV-associated cognitive impairment) (McGuire et al., 2016), parasite (*Plasmodium*, CM) (Lackner et al., 2008), and prion (Klein et al., 2001) infections, while it may be protective during the initial phase of prion disease (Flores-Langarica et al., 2009). Jointly these findings indicate the deleterious consequences of overactivation of the classical pathway. The lectin pathway is protective against herpes simplex virus encephalitis by inhibiting viral dissemination into the CNS (Bibert et al., 2019). While C3-mediated pathogen clearance is beneficial in the periphery (Mehlhop et al., 2005; Brooke et al., 2012; Sun et al., 2016), C3d deposition on synaptic terminals may facilitate synaptic loss in WNV infection (Vasek et al., 2016). Likewise, the net outcome of downstream complement activation seems to be context-dependent. C5 is important for fungal clearance in the vasculature (Sun et al., 2016) and inhibits fungal dissemination into the CNS (Tuite et al., 2005). In contrast, in the *Plasmodium* infection, C5 deficiency is protective against CM (Patel et al., 2008; Ramos et al., 2011; Buckingham et al., 2014). Inhibition of MAC formation was also protective against CM (Ramos et al., 2011), confirming the crucial role of terminal complement pathway in CM pathogenesis. C3aR was up-regulated in the brain upon viral infection (Libbey et al., 2017) and C3aR deficiency was protective against viral infection-induced synaptic loss (Vasek et al., 2016). C5aR deficiency was also protective against CM development (Patel et al., 2008; Kim et al., 2014; McDonald et al., 2015), as well as bacterial meningitis (Woehrl et al., 2011). However, neither C3aR deficiency nor C5aR deficiency were protective against prion-induced neuropathology (Carroll et al., 2018), indicating pathogen-specific roles of anaphylatoxin receptors in the CNS.

CD46, a negative regulator of the classical and lectin pathways, aids in the dissemination of bacteria (*N. meningitidis*) (Johansson et al., 2003, 2005) and viruses [Measles virus (Rall et al., 1997; Manchester et al., 1999; Oldstone et al., 1999; Evlashev et al., 2000) and HHV-6 (Cassiani-Ingoni et al., 2005)] into the CNS, leading to meningitis and encephalitis, respectively. While the use of CD46 by these pathogens could be an evasion strategy from the classical or lectin pathway-mediated clearance, CD46-dependent facilitation of immune cell infiltration into the CNS (Manchester et al., 1999; Oldstone et al., 1999) and cell-cell fusion of infected lymphocytes and glial cells (Cassiani-Ingoni et al., 2005) suggest higher mechanistic complexity of the interactions between complement and infectious agents. FH, a negative regulator of the alternative pathway, is also engaged in the dissemination of pathogens in bacterial (*S. suis*) (Kong et al., 2017) and prion infections (Kane et al., 2017), indicating that these infectious agents manipulate the alternative complement pathway for their propagation in the brain. The fact that a viral RCA homolog plays a facilitatory role in viral dissemination via the inhibition of C3-dependent host defense (Kapadia et al., 2002) suggests that infectious agents have evolved diverse molecular mechanisms to escape complement-mediated killing.

## Discussion

### Potential Link Between Infection-Induced Complement Activation and Dementia

**FIGURE 3:**
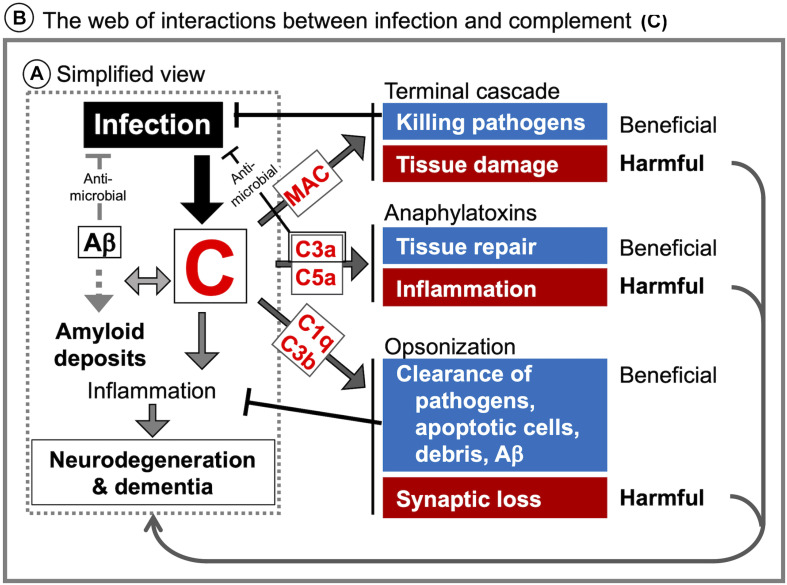
Infection-induced complement activation in the CNS and potential links to neurodegeneration and dementia. **(A)** The most simplified view of how CNS infection can lead to neurodegeneration and dementia is enclosed in the dotted rectangle. Infection-induced complement activation products may contribute to neurodegeneration and dementia via interaction with amyloid deposits and enhanced neuroinflammation. **(B)** A more detailed schematic representation of specific interactions between infectious agents and the complement system as a potential mechanistic link between CNS infection, neurodegeneration and dementia. The consequences of complement activation may be context-dependent. For example, MAC plays a role in pathogen elimination but can also contribute to tissue damage. C3a and C5a can support tissue repair and adaptive neural plasticity, but also promote potentially detrimental inflammation and leukocyte recruitment. Opsonization and phagocytosis are needed for clearance of pathogens, but the same mechanisms are involved in synapse elimination as the first step in neurodegeneration.

Considering the link between cognitive impairment and aberrant complement activation in the CNS (Aiyaz et al., 2012; Rubio-Perez and Morillas-Ruiz, 2012), the most simplified view is that CNS infection could lead to dementia via the complement activation (Figure 3A: dotted rectangle). However, as discussed above, the mechanistic links between infectious agents, complement, and neurodegeneration are far more complex. Due to the multiple neurodevelopmental and homeostatic functions that the complement system and its individual components play in the brain (Figure 1), complement activation by infectious agents can cause an imbalance in the intricate web of interactions between complement proteins and the nervous system with deleterious consequences for CNS tissue integrity and function (Figure 3B: solid rectangle). This could explain the unpredictable and sometimes inconsistent observations in different studies in human patients as well as in animal models of CNS infection. The multiple functions of the complement system in the CNS together with the complexity of immune responses to infection call for well-informed and highly selective approaches when manipulating the complement system for therapeutic benefit. For example, inhibiting the down-stream of complement cascade (C5 and MAC) and/or C5aR could protect the brain from *Plasmodium* infection-induced tissue damage (Patel et al., 2008; Ramos et al., 2011; Buckingham et al., 2014; Kim et al., 2014), whereas inhibition restricted to the upstream components (C1q, C2, and FB) could be beneficial in prion diseases (Klein et al., 2001), as shown in animal models. However, these studies were conducted using constitutive inactivation of specific genes. As the complement system plays multiple roles in the CNS, roles which are distinct from those in the periphery, it is imperative to investigate CNS-specific roles of the complement system in the context of an infection by a specific pathogen. Furthermore, while complement proteins are locally produced in the brain, it is less clear which cell types are responsible, how complement protein production and responses are regulated, and how are these processes influenced by various stimuli under physiological and pathological conditions, including CNS infection. Of particular interest in this respect are the potential functions of intracellular C3 (Elvington et al., 2016), not only with regard to the interactions between infectious agents and the brain-resident cells but also the role of intracellular C3 in neurodegeneration. Considering the diverse roles of glial cells (astrocytes and microglia, the brain-resident macrophages) in the maintenance of CNS homeostasis, further studies are required to elucidate how infectious agents modulate complement generation in glial cells and how, in turn, it might affect the phenotypes of these cells.

## Conclusion

Of the approximately 50 million people with dementia worldwide, about 50% live in low- and middle-income countries (WHO, 2019). Considering higher risk of infection and infection-induced brain disorders in those countries (Fernandes and Caramelli, 2019; Singh et al., 2020), understanding of the potential mechanistic links between infection and neurodegenerative disorders including dementia, will inform the development of preventive and therapeutic strategies. While amyloidogenesis is widely accepted as the underlying cause of AD and other forms of neurodegenerative diseases, Aβ-targeting drug development has been so far unsuccessful (Robakis, 2020). Given that the presence of amyloid plaques does not necessarily indicate cognitive decline (Skaper, 2012; Robakis, 2020), alternative culprits and theories are needed. Indeed, there is a growing body of evidence showing that innate immunity and neuroinflammation play a pivotal role in dementia development (Leng and Edison, 2020).

Separated from the other parts of the body by the BBB and CSF barrier, the CNS is uniquely protected by special immune components, including the complement system and glial cells. While locally produced complement components play crucial homeostatic roles in the CNS, dysregulated complement activity could be detrimental to the function of the CNS. As the key element of brain-resident innate immunity, the complement system plays both protective and destructive roles in neurodegeneration (Kolev et al., 2009). Whereas aberrant complement activation is frequently associated with neuroinflammation and neurodegenerative disorders, the complement system exerts homeostatic functions via eliminating apoptotic cells, cell debris, and toxic substances, and supports plasticity and tissue repair. A broad range of infectious agents can potentially interact with the complement system in the brain and the net outcome of these interactions appears to depend on the specific pathogens, the target components, and the mode of interaction. It is therefore conceivable that pathogen-triggered and persistent activation of the complement system represents the missing mechanistic link between infection, in particular infection affecting the CNS, and neurodegeneration. We are fully aware that the simplified view presented in Figure 3 does not capture every aspect of the involvement of the complement system in infection-induced neuropathology and neurodegeneration, but only highlights the key components of the interplay between pathogens and complement activation induced–neurodegenerative processes that may lead to dementia. While the specific mechanistic links between CNS infection, neurodegeneration and dementia are still unclear, the involvement of the complement system and the pathogen- and disease stage-specific roles of the individual complement proteins warrant further intense investigation.

## Author Contributions

NS wrote the first draft of the manuscript. MP and WK critically assessed the content. NS and MP jointly revised and finalized the manuscript. All the authors contributed to the article and approved the submitted version.

## Conflict of Interest

The authors declare that the research was conducted in the absence of any commercial or financial relationships that could be construed as a potential conflict of interest.

## Publisher’s Note

All claims expressed in this article are solely those of the authors and do not necessarily represent those of their affiliated organizations, or those of the publisher, the editors and the reviewers. Any product that may be evaluated in this article, or claim that may be made by its manufacturer, is not guaranteed or endorsed by the publisher.
